# A Cooperative Search and Coverage Algorithm with Controllable Revisit and Connectivity Maintenance for Multiple Unmanned Aerial Vehicles

**DOI:** 10.3390/s18051472

**Published:** 2018-05-08

**Authors:** Zhong Liu, Xiaoguang Gao, Xiaowei Fu

**Affiliations:** School of Electronics and Information, Northwestern Polytechnical University, Xi’an 710072, China; cxg2012@nwpu.edu.cn (X.G.); fxw@nwpu.edu.cn (X.F.)

**Keywords:** multi-UAVs, search and coverage, digital pheromone, distributed receding horizon optimizing, collision avoidance, connectivity maintenance, minimum spanning tree, potential field

## Abstract

In this paper, we mainly study a cooperative search and coverage algorithm for a given bounded rectangle region, which contains several unknown stationary targets, by a team of unmanned aerial vehicles (UAVs) with non-ideal sensors and limited communication ranges. Our goal is to minimize the search time, while gathering more information about the environment and finding more targets. For this purpose, a novel cooperative search and coverage algorithm with controllable revisit mechanism is presented. Firstly, as the representation of the environment, the cognitive maps that included the target probability map (TPM), the uncertain map (UM), and the digital pheromone map (DPM) are constituted. We also design a distributed update and fusion scheme for the cognitive map. This update and fusion scheme can guarantee that each one of the cognitive maps converges to the same one, which reflects the targets’ true existence or absence in each cell of the search region. Secondly, we develop a controllable revisit mechanism based on the DPM. This mechanism can concentrate the UAVs to revisit sub-areas that have a large target probability or high uncertainty. Thirdly, in the frame of distributed receding horizon optimizing, a path planning algorithm for the multi-UAVs cooperative search and coverage is designed. In the path planning algorithm, the movement of the UAVs is restricted by the potential fields to meet the requirements of avoiding collision and maintaining connectivity constraints. Moreover, using the minimum spanning tree (MST) topology optimization strategy, we can obtain a tradeoff between the search coverage enhancement and the connectivity maintenance. The feasibility of the proposed algorithm is demonstrated by comparison simulations by way of analyzing the effects of the controllable revisit mechanism and the connectivity maintenance scheme. The Monte Carlo method is employed to validate the influence of the number of UAVs, the sensing radius, the detection and false alarm probabilities, and the communication range on the proposed algorithm.

## 1. Introduction

Recently, multiple UAVs have received more and more attention for their accomplishments in both military and civil applications. Cooperative search and coverage is one major application of multiple UAVs equipped with sensors [1], such as camera, radar, and sonar. The goal of cooperative search and coverage is to control multiple UAVs to find several unknown ground targets scattered in a given surveillance region, while maximally reducing the uncertainty of the environment and minimizing the search time [2].

In the cooperative search and coverage problem, there are two main technical issues that should be considered [3]. (1) The environment representation and update. This focuses on how to represent targets existence and uncertainty in the environment, and how to treat sensor observation results as evidence to update the knowledge of the environment so that the UAVs’ belief can reflect the true existence or absence of the targets within areas of the surveillance region; (2) search path planning. This focuses on how develop cooperative control methods that enable UAVs to move in such a way as to maximize the possibility of finding targets or minimize the uncertainty in the environment. Extensive studies have been carried out on these two key issues.

The environment representation is the primary problem of cooperative search and coverage. In general, a common method is to make the whole surveillance area become smaller cells, and each cell is associated with values, such as probability or uncertainty, thereby constituting a map for the search region. Each UAV maintains a map of the surveillance region that serves as the UAV’s knowledge about the state of the region. There are several types of the map, such as the occupancy map [4,5,6], the target probability map [7,8,9], the uncertainty map [10,11,12], and so on. In fact, these maps can collectively be called the cognitive map, which is used to represent the environment and is incrementally updated based on the new sensor observations.

The continuous updating of the cognitive map reflects the collecting and processing of new sensory information about the environment for the UAVs. However, each UAV can only obtain local sensory information about the whole surveillance region due to its limited sensing range. In addition, considering the non-ideal sensor performance, there are a great variety of errors and uncertainties on the sensory information from the UAVs. Therefore, the sensory information from multiple UAVs needs to be combined through some update and fusion schemes so that the best knowledge of the environment can be obtained. Most available update and fusion schemes are broadly based on Bayesian theory [2,13,14] and Dempster-Shafer theory [15]. However, few of the existing update and fusion methods can guarantee that all individual cognitive maps can be converged to the same one that reflects the true target existence status of each cell in the whole surveillance region.

Search path planning is an important problem for efficient search and coverage by a team of UAVs. It is concerned with cooperative controlling of the movements of multi-UAVs in order to guarantee optimal search paths that maximize the possibility of finding targets and minimize the uncertainty in the environment. Different authors have developed several search path planning methods, such as reinforcement learning [15], potential field [16], group dispersion pattern [17], intelligence algorithm [18], dynamic programming [19], gradient optimization [20], mixed integer linear programming [21], Voronoi partitioning [22,23], and receding horizon optimization (RHO) [24,25,26]. In [22,23], the convex region is partitioned into Voronoi cells so that there is only one agent in each Voronoi cell according to the position of the agents. Therefore, the distributed multi-agents coverage control problem is converted into the coverage of Voronoi cell for single agent. Then, a gradient descent control law is designed to continually drive the agents toward the centroids of their Voronoi cells. In this way, the whole convex region can be covered by multi-agents without violating the collision constraint.

For the reason of the fact that the RHO could effectively handle the dynamic changes of the environment and constrain movements of UAVs, it is used to solve the cooperative search and coverage problems. Reference [24] presents a receding horizon cooperative search algorithm that jointly optimizes paths and sensor orientations for a team of UAVs searching for a mobile target. In reference [25], a receding horizon, motion-planning algorithm is used to obtain the optimal search path in the given horizon. In reference [26], the distributed model predictive control method is presented to solve the cooperative search moving targets problem. In addition, the attractant and repulsion pheromones are introduced to improve the effective cooperation between UAVs.

Although these above methods have been verified to be effective for the multi-UAVs cooperative search and coverage problems, they lack a controllable revisit mechanism to guide the UAVs to revisit sub-areas with large target probability or high uncertainty, so that the capability to capture the target and the efficiency of coverage are low.

Furthermore, the sufficient information exchange and sharing in the whole team of UAVs is quite essential for cooperative search and coverage, and thus it is required that the UAVs could maintain a connected communication network. Connectivity maintenance has been applied in the multiple agents system. For example, in [27], a decentralized power iteration algorithm is designed to estimate the connectivity of the multi-agents network. Then, a gradient control law for each agent is proposed to maintain global connectivity. Reference [28] uses the potential fields to drive the agents to a full connected configuration while avoiding collisions with each other. However, these methods cannot be applied directly in the cooperative search and coverage problem in this paper. These methods often generate a full connected topology with dense communication links, which may largely restrict the motion of the UAVs and jeopardize the efficiency of cooperative search and coverage. In fact, adding communication links improves the network connectivity, but the movement of the UAVs will be extremely limited. Disconnecting links may destroy the network connectivity, but on the other hand, that will reduce UAV movement restrictions and provide more freedom to explore wider areas and increase the efficiency of searching and covering [25]. Thus, the tradeoff between the search coverage enhancement and the connectivity preservation should be considered, which aims to maintain the connected network while relaxing the motion constraints on the UAVs as possible.

In this paper, we mainly study cooperative search and coverage for a given bounded rectangle region, which contains several unknown stationary targets, by a team of UAVs with non-ideal sensors and a limited communication range. The goal of mission is to minimize the search time, while gathering more information about the environment and finding more targets. During the mission process, the collision avoidance and network connectivity constraints must be guaranteed.

The main contributions to this paper contain three aspects. (1) A distributed update and fusion scheme for the cognitive map is proposed. We prove that this update and fusion scheme can guarantee that all individual cognitive maps converge to the same one that reflects the true target existence status of each cell in the search region; (2) considering the revisit requirement for the sub-areas in the search region, we develop a controllable revisit mechanism based on a digital pheromone. This mechanism can control the UAVs to revisit sub-areas with large target probability or high uncertainty; (3) aiming to achieve the tradeoff between the search coverage enhancement and the connectivity maintenance, a connectivity maintenance control strategy based on the minimum spanning tree (MST) topology optimization is presented. The network topology is optimized using the MST, and only the communication links in the MST topology are maintained. Thus, the UAVs remove the redundant links without violating the global connectivity condition, and hence obtain more freedom to be dispersed for the search and coverage enhancement.

The structure of this paper is organized as follows. In Section 2, the mission scenario, together with the problem formulation, is provided. In Section 3, as the representation of the environment, cognitive maps that include the target probability map (TPM), the uncertain map (UM), and the digital pheromone map (DPM) are constituted. We design an update and fusion scheme for the individual cognitive maps such that they are all converge to the same one that reflects the true environment. We also develop a controllable revisit mechanism based on DPM. This mechanism can command the UAVs to revisit some important areas where they have a high target probability or that they have not explored for a long time. In Section 4, we propose our path planning algorithm for multi-UAVs cooperative search and coverage operation. The path planning is performed in a distributed fashion. Each UAV solves local rolling time domain optimization problem, and obtains its own optimal path to search and cover the surveillance region. In this path-planning algorithm, the movement of UAVs is restricted by the potential fields to satisfy the collision avoidance and connectivity maintenance constraints. In addition, the tradeoff between the coverage enhancement and the connectivity maintenance is achieved using the MST topology optimization strategy. We present simulation and experimental results in Section 5, followed by summary and conclusions in Section 6.

## 2. Problem Formulations

**Figure 1:**
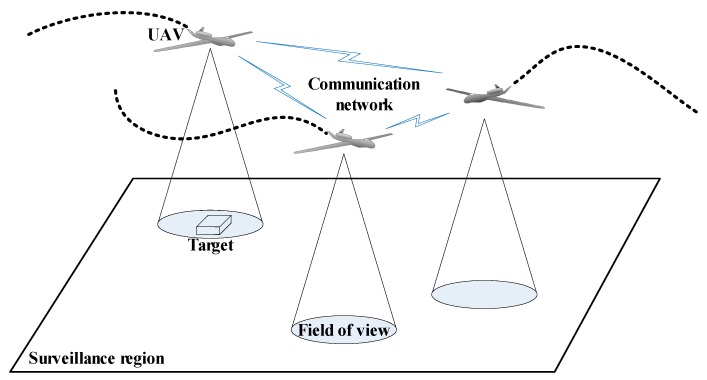
Target search by multiple UAVs.

As shown in Figure 1, there is a team of UAVs (*A_i_*, *i* = 1, 2, …, *N*) performing cooperative search and coverage mission in an unknown surveillance region that contained several stationary ground targets (*T_j_*, *j* = 1, 2, …, *M*). In unknown environment, for the UAVs, the targets number and their locations are unknown a priori. The UAVs need to use on-board sensors (e.g., cameras) to observe some areas of the environment, so that the UAVs can incrementally obtain knowledge of the environment and find targets. The decision on where to explore next is driven by the objective to increase the chance of finding targets or reducing the uncertainty in the environment. For this purpose (1) we need to design a **cognitive map** as the environment representation, to represent targets existence and uncertainty in the environment. In other words, each UAV maintains a cognitive map of the whole surveillance region that serves as the UAV’s knowledge of the environment; (2) an **update and fusion scheme** need to be designed to guarantee all cognitive maps converge to the same one that reflects the targets’ true existence or absence in each cell of the search region. As the UAVs move around and observe sub-areas of the region, the corresponding cells of the cognitive maps are updated to incorporate the information gained through the on-board sensors, as well as the communication network. The updated cognitive maps should be same and could reflect the true environment; (3) we need to design a **distributed search and coverage control algorithm** that plans the optimal paths for the UAVs to follow to search and coverage the surveillance region, while guarantying the constraints of the collision avoidance and connectivity preservation.

### 2.1. The Description of Search Environment

**Figure 2:**
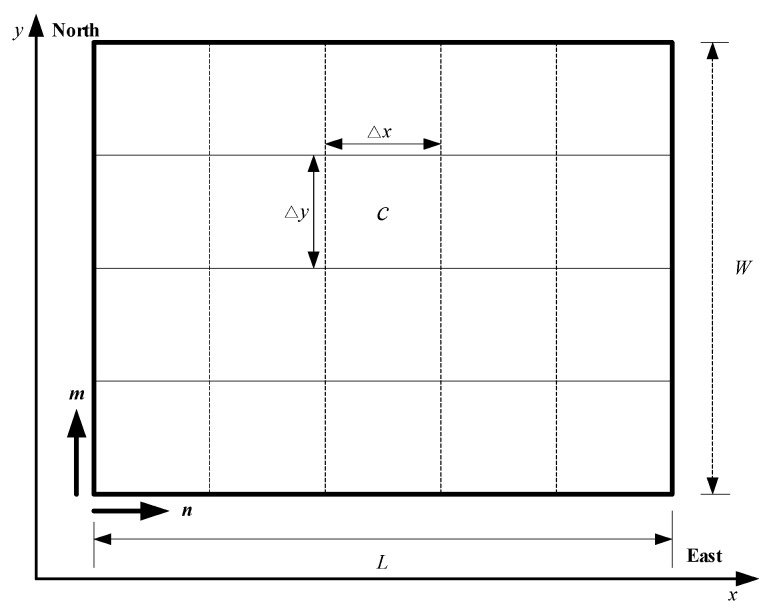
A rectangular surveillance region is uniformly divided into *L_x_* × *L_y_* cells of the same size.

As shown in Figure 2, an *L* × *W* rectangular region ***Ω*** is uniformly divided into *L_x_* × *L_y_* cells of the same size. The cell that is located in the *m*-th row and *n*-th column is identified by its identity number *c* = *m* + (*n* − 1) × *L_x_*, *c* ∊ {1, 2, …, *L_x_* × *L_y_*}. Δ*x* and Δ*y* denote the length and width of the cells, respectively. Δ*x* and Δ*y* may be chosen as the flight distance of the UAV in a time step with the constant cruising speed. Each cell is located with its center ***μ****_c_* = [*x_c_*, *y_c_*]^T^, in which *x_c_* and *y_c_* are coordinated with its center. *ζ_c_* ∊ {0, 1} indicates whether a target exists in cell c or not, i.e., *ζ_c_* = 1 indicates a target is present in cell *c*, and *ζ_c_* = 0 indicates no target is present in that cell. To better describe the problem, it assumes that (i) there is, at most, one target in each cell and (ii) there are no threats and obstacles in the surveillance region.

### 2.2. Simplified Dynamic Model of UAV

We assume that the UAVs move on a fixed plane above the surveillance region. Let ***x****_i_*(*k*) = [***μ****_i_*(*k*), *ψ_i_*(*k*)]^T^ denote the state of *A_i_* at time *k*. ***μ****_i_*(*k*) = [*x_i_*(*k*), *y_i_*(*k*)]^T^ represents the position of *A_i_*, in which *x_i_*(*k*) and *y_i_*(*k*) are the planar coordinates of its projection onto the surveillance region. *ψ_i_*(*k*) is the heading angle. The two-dimension motion of UAV in a horizontal plane is analyzed, and the kinematics of the UAV are

(1) $$\{\begin{array}{c} x_{i}(k+1)=x_{i}(k)+\mathrm{In}[\frac{v_{c}\cdot \Delta t\cdot \cos\psi_{i}(k)}{\Delta x}]\\ y_{i}(k+1)=y_{i}(k)+\mathrm{In}[\frac{v_{c}\cdot \Delta t\cdot \sin\psi_{i}(k)}{\Delta y}] \end{array}$$

In Equation (1), *v_c_* represents the constant cruising speed, and Δ*t* represents the time step. Operator In [·] indicates a rounding operation that maps the flight distance of *A_i_* over a time step Δ*t* to the cell index increments (Δ*m*, Δ*n*) in surveillance region.

For simplicity, it is assumed that the UAV is equipped with an autopilot that holds constant altitude and ground speed. We only need to design guidance inputs to this low-level autopilot system for target searching. In this paper, the design guidance inputs are *u_i_*(*k*) = *ψ_i_*(*k*), which are constrained by the dynamic limits of UAV, i.e., the turning rate Δ*ψ_i_*(*k*) ∊ [−Δ*ψ*_max_, Δ*ψ*_max_]. According to Equation (1), the dynamics of the UAVs can be described as follows. At each Δ*t*, *A_i_* can only move from the current cell to the neighboring cells, due to the constraints of maneuverability. More specifically, there are eight possible flight orientations defined as *ψ_i_*(*k*) ∊ {1(east), 2(northeast), 3(north), 4(northwest), 5(west), 6(southwest), and 7(south)}. Due to its physical curvature radius constraints, the UAV can only change its orientation at most once in a Δ*t*. In this case, *A_i_* has only three possible orientation choices (turn left, go straight, or turn right, denoted by Δ*ψ_i_*(*k*) ∊ {*l*(left), *s*(straight), *r*(right)}) for the next time step. Thus, the maximum turning angle is Δ*ψ*_max_ = 45°.

### 2.3. Communication Model

In this paper, we only consider the limited communication range and ignore the bandwidth limitation, the communication delay, and the interruption that will be the future research direction extending the current work. Thus, two UAVs can directly exchange information if the distance between them is no more than the communication range *R*_c_. The network topology of the l UAVs at time *k* can be modeled as an undirected graph ***G***(*k*) = (***V***, ***E***(*k*)). ***V*** = {*A*_1_, …, *A_N_*} is the vertices set and ***E***(*k*) = {(*A_i_*, *A_j_*)| (*A_i_*, *A_j_*) ∊ ***V***; ||***μ****_i_*(*k*) − ***μ****_j_*(*k*)|| ≤ *R*_c_} is the edge set, in which ||·|| denotes the 2-norm for vectors. Let ***N****_i_*(*k*) = {*A_j_*|||***μ****_i_*(*k*) − ***μ****_j_*(*k*)|| ≤ *R*_c_; *j* = 1, …, *N*, and *j* ≠ *i*} denote the set of neighbors of *A_i_* at time *k*. The adjacency matrix can be expressed as

(2) $$A(k)=[a_{ij}]=\{\begin{array}{l} \omega_{ij},(A_{i},A_{j})\in E(k)\\ 0,\mathrm{otherwise} \end{array}$$

in which *ω_ij_* > 0 is the weight of the wireless link (*A_i_*, *A_j_*). In this paper, *ω_ij_* is defined as

*ω_ij_* = (*d_ij_*/1000)^3^ (3)

in which *d_ij_* = ||***μ****_i_*(*k*) − ***μ****_j_*(*k*)|| is the distance between *A_i_* and *A_j_*. From Equation (3), we can see that a greater between *A_i_* and *A_j_* results in the larger weight of the link (*A_i_*, *A_j_*). Then, the Laplacian matrix can be expressed as

(4) $$L(k)=[l_{ij}]=\{\begin{array}{l} \sum_{j=1,j\neq i}^{N}a_{ij},i=j\\ -a_{ij},i\neq j \end{array}$$

***G***(*k*) is **full connected** if direct communication exists between every two vertices of the graph. ***G***(*k*) is **connected** if a sequence of edges (a route) exists for any two vertices; otherwise, ***G***(*k*) is **unconnected**. The information sharing is quite indispensable in the cooperative search and coverage mission, and thus the network connectivity must be preserved. Let 0 = *λ*_1_(*k*) ≤ *λ*_2_(*k*) ≤ … ≤ *λ_N_*(*k*) be the ordered eigenvalues of the Laplacian matrix ***L***(*k*). According to the algebraic graph theory, if and only if *λ*_2_(*k*) > 0, the graph ***G***(*k*) is connected. The second smallest eigenvalue of ***L***(*k*), *λ*_2_(*k*), is also called as the algebraic connectivity of the graph.

## 3. Cognitive Map

One of the most important aspects of search and coverage mission is to create a representation of the environment that contains information about targets existence and uncertainty within each cell. In this section, the notion of cognitive map is used as the environmental representation. We firstly construct the target probability map (TPM), which is used to describe the probabilities of the cells being occupied by a target. Next, the other two maps are introduced based on TPM. One is uncertainty map (UM), and it can describe the uncertainty degree of the environment. The other is digital pheromone map (DPM), which is mainly used to establish the controllable revisit mechanism. Integrating TPM, UM, and DPM, the cognitive map can be designed.

### 3.1. The Target Probability Map

If prior intelligent information obtained is not accurate, UAV can not absolutely know the distribution of the targets. The TPM is used to describe target existing probability of each cell. The target existing probability *p_c_*(*k*) ∊ [0, 1] is modeled as a Bernoulli distribution, i.e., *p_c_*(*k*) = *P*(target present in cell *c* at time *k*), (1 − *p_c_*(*k*)) = *P*(no target present in cell *c* at time *k*). The higher *p_c_*(*k*) is, the more likely the UAV believes that a target is in cell *c*. *p_c_*(*k*) = 0.5 indicates that the UAV has no knowledge about target existence in cell *c*, because the probability that a target is present is equal to the probability that no target is present in cell *c*. Based on the above notations, the TPM is defined as

***M****_i_*_,TPM_(*k*) = {*p_i,c_*(*k*)|*c* ∊ ***Ω***} (5)

In the mission processing, each UAV maintains its own TPM. The knowledge of each UAV on target existence state in cell *c* is based on its sensor observations and the shared information from the neighboring UAVs by communication. So, the update of individual TPM has two stages: update TPM based on sensor observations and update TPM based on shared information.

#### 3.1.1. Update TPM Based on Sensor Observations

It is assumed that *A_i_* takes observations via a downward-facing camera. The field of view (FoV) is a circle with sensing radius *R*_s_ that covers some cells in the surveillance region ***Ω***. Therefore, at time *k*, the set of the covered cells inside the FoV of *A_i_* denotes ***Φ****_i,k_*

***Φ****_i,k_* = {*c* ∊ ***Ω***: ||***μ****_c_* − ***μ****_i_*(*k*)|| ≤ *R*_s_} (6)

The sensor observations about cell *c* at time *k* are defined as *Z_i,c,k_* ∊ {0, 1}, in which *Z_i,c,k_* = 1 indicates “target detection” and *Z_i,c,k_* = 0 indicates “non-target detection”. However, the sensor is non-idear. The performance of the sensor can be described by detection probability *p*_d_ and false alarm probability *p*_f_, i.e., *P*(*Z_i,c,k_* = 1|*ζ_c_* = 1) = *p*_d_ and *P*(*Z_i,c,k_* = 1|*ζ_c_* = 0) = *p*_f_. We assume that, for all cells and UAVs, *p*_d_ and *p*_f_ are constant and known prior.

According to the sensor observations *Z_i,c,k_*, the *p_i,c,k_* ≜ *p_i,c_*(*k*) is update via following rule, which is based on Bayesian theory

(7) $$p_{i,c,k}=\frac{p(Z_{i,c,k}|\zeta_{c}=1)p_{i,c,k-1}}{p(Z_{i,c,k}|\zeta_{c}=1)p_{i,c,k-1}+p(Z_{i,c,k}|\zeta_{c}=0)(1-p_{i,c,k-1})}\;=\{\begin{array}{ll} \frac{p_{d}p_{i,c,k-1}}{p_{d}p_{i,c,k-1}+p_{f}(1-p_{i,c,k-1})}, & c\in \Phi_{i,k}\;\mathrm{and}\;Z_{i,c,k}=1\\ \frac{(1-p_{d})p_{i,c,k-1}}{\left(1-p_{d})p_{i,c,k-1}+(1-p_{f})(1-p_{i,c,k-1}\right)}, & c\in \Phi_{i,k}\;\mathrm{and}\;Z_{i,c,k}=0\\ p_{i,c,k-1}, & c\notin \Phi_{i,k} \end{array}$$

By introducing the nonlinear transformation that described in Equation (8), the update rule is rewritten as shown in Equation (9).

(8) $$Q_{i,c,k}≜\ln(\frac{1}{p_{i,c,k}}-1)$$

(9) $$Q_{i,c,k}=Q_{i,c,k-1}+\upsilon_{i,c,k};\upsilon_{i,c,k}≜\{\begin{array}{l} \ln\frac{p_{f}}{p_{d}},c\in \Phi_{i,k}\;\mathrm{and}\;Z_{i,c,k}=1\\ \ln\frac{1-p_{f}}{1-p_{d}},c\in \Phi_{i,k}\;\mathrm{and}\;Z_{i,c,k}=0\\ 0,\;c\notin \Phi_{i,k} \end{array}$$

From Equation (9), we can see that the evolution of *Q_i,c,k_* depends on the number of detections that is taken over cell *c* up to time *k*, which is denoted as *m_i,c,k_*. In fact, *m_i,c,k_* → +∞, *p_i,c,k_* → 0 if no target is present in cell *c*, and *p_i,c,k_* → 1 if a target is present in cell *c*. The symbol “→” indicates “approaches”. It means that the converged TPM, ***M***_*i*,TPM_(*k*), can reflect the true existence and absence of the targets. To prove this view, the convergence property of the updating method (Equation (9)) is analyzed in Theorem 1.

**Theorem** **1.** *Given the initial prior TPM 0 < p_i,c,0_ < 1 for A_i_., and 0 < p_f_ < 0.5 < p_d_ < 1, the following conclusions hold by implementing the updating rule in Equation (9).*

- *If ζ_c_ = 1, which indicates a target is present in cell c, as m_i,c,k_ → +∞, then Q_i,c,k_ → −∞ (i.e., p_i,c,k_ → 1) and (Q_i,c,k_/m_i,c,k_) → p_d_ln(p_f_/p_d_) + (1 − p_d_)ln(1 − p_f_/1 − p_d_);*
- *If ζ_c_ = 0, which indicates no target is present in cell c, as m_i,c,k_ → +∞, then Q_i,c,k_ → +∞ (i.e., p_i,c,k_ → 0), and (Q_i,c,k_/m_i,c,k_) → p_f_ ln(p_f_/p_d_) + (1 − p_f_)ln(1 − p_f_/1 − p_d_).*

The proof of Theorem 1 is seen in Appendix A. From Equation (9), we can find several interesting properties. If 0 < *p*_f_ < 0.5 < *p*_d_ < 1, which means that the sensor could provide useful information, then *p_i,c_*(*k*) > *p_i,c_*(*k* + 1) if a target is present in cell *c* and *p_i,c_*(*k*) < *p_i,c_*(*k* + 1) if no target is present in cell *c*. Therefore, the upper bound *p*_max_ and the lower bound *p*_min_ of the target existing probability are introduced. If *p_i,c,k_* ≥ *p*_max_, the UAV has obtained enough evidence to support its belief in a target existing in cell *c*. If *p_i,c,k_* ≤ *p*_min_, the UAV confirms that no target exists in cell *c*. In order to confirm whether there is a target in cell *c* or not, the UAVs needs to detect cell *c* several times to update the target existence probability to approach the upper bound *p*_max_ or the lower bound *p*_min_. Theorem 2 shows how to estimate the minimum number of observations required in a given cell *c*.

**Theorem** **2.** *Given the initial prior TPM 0 < p_i,c,0_ < 1 for A_i_, and 0 < p_f_ < 0.5 < p_d_ < 1.*

- *If a target is present in cell c, the minimum number of observations* $m_{\mathrm{avg}}^{+}$ *required in cell c to satisfy the condition p_i,c,k_ ≥ p_max_ is estimated as*
  (10) $$m_{\mathrm{avg}}^{+}\geq \frac{\ln\left[\frac{p_{i,c,0}(1-p_{\max})}{p_{\max}(1-p_{i,c,0})}\right]}{p_{d}\ln\frac{p_{f}}{p_{d}}+(1-p_{d})\ln\frac{1-p_{f}}{1-p_{d}}}$$
- *If no target is present in the cell c, the minimum number of observations* $m_{\mathrm{avg}}^{-}$ *required in cell c to satisfy the condition p_i,c,k_ ≤ p_min_ is estimated as*
  (11) $$m_{\mathrm{avg}}^{-}\geq \frac{\ln\left[\frac{p_{i,c,0}(1-p_{\min})}{p_{\min}(1-p_{i,c,0})}\right]}{p_{f}\ln\frac{p_{f}}{p_{d}}+(1-p_{f})\ln\frac{1-p_{f}}{1-p_{d}}}$$

The proof of Theorem 2 is seen in [3]. We can see that the sensor performance is better, e.g., when the detection probability *p*_d_ and the lower the false alarm probability *p*_f_ are larger, the minimum number of observations required $m_{\mathrm{avg}}^{+}$ or $m_{\mathrm{avg}}^{-}$ is smaller. This means that the better the performance of the sensor, the faster the TPM converges.

#### 3.1.2. Update TPM Based on Shared Information

First, according to the sensor observations *Z_i,c,k_*, each UAV *A_i_* at time *k* updates its own TPM using Bayesian rule in Equation (9).

*H_i,c,k_* = *Q_i,c,k_* + *v_i,c,k_* (12)

Then, each UAV *A_i_* exchanges the updated TMP *H_i,c,k_* to neighboring UAVs, and updates its own TPM again (map fusion) by following consensus protocol

(13) $$Q_{i,c,k}=\sum_{j=1}^{N}\omega_{i,j,k}H_{j,c,k}$$

in which *ω_i,c,k_* = 1 − (|***N****_i_*(*k*)|/*N*), *ω_i_*_,*j*,*k*_ = (1/*N*) for *j* ∊ ***N****_i_*(*k*), and *ω_i,c,k_* = 0 for *j* ∉ ***N****_i_*(*k*). ***N****_i_*(*k*) is the set of neighbors of *A_i_* at time *k*.

Similar to the Theorem 1, the rationality and the convergence property of the distributed update and fusion method in Equation (13) is analyzed in Theorem 3.

**Theorem** **3.** *Given the initial prior TPM 0 < p_i,c,0_ < 1 for A_i_., and 0 < p_f_ < 0.5 < p_d_ < 1, if the network topology **G**(k) of the UAVs is connected at all times, the following conclusions hold by implementing the distributed update and fusion scheme in Equation (13).*

- *If ζ_c_ = 1, which indicates a target is present in the cell c, as m_c,k_ → +∞, then Q_i,c,k_ → −∞ (i.e., p_i,c,k_ → 1) and (Q_i,c,k_/m_c,k_) →* *(p_d_* */N)ln(p_f_/p_d_) +* *((1 − p_d_)* */N)ln(1 − p_f_/1 − p_d_);*
- *If ζ_c_ = 0, which indicates no target is present in the cell c, as m_c,k_ → +∞, then Q_i,c,k_ → +∞ (i.e., p_i,c,k_ → 0), and (Q_i,c,k_/m_c,k_) → (p_f_ /N)ln(p_f_/p_d_) + ((1 − p_f_) /N)ln(1 − p_f_/1 − p_d_).*

*in which m_c,k_ =* $\sum_{i=1}^{N}m_{i,c,k}$ *represents the total number of detections that taken over the cell c up to time k by the whole team of UAVs.*

The proof of Theorem 3 is seen in Appendix B. Theorem 3 gives two important conclusions, as follows

1. As *m_c,k_* → +∞, the converged TPM, ***M***_*i*,TPM_(*k*), *i* = 1, 2, …, *N*, which is updated according to the distributed update and fusion scheme in Equation (13), can reflect the true existence and nonexistence of the targets. Thus, the rationality of the distributed update and fusion scheme in Equation (13) can be proven.
2. The relationship between the average detected rate of the cells and the convergence speed of the TPM is explicated in Theorem 3. For example, if a target is present in the cell *c*, as *m_c,k_* → +∞
  (14) Qi,c,kmc,k=Qi,c,kkmc,kNk→pdlnpfpd+(1−pd)ln1−pf1−pd≜a
  in which (*m_c,k_*/(*Nk*)) represents the global average detected rate of the cell *c* by the UAVs. Equation (14) shows that the speed of *Q_i,c,k_* approaching −∞ or +∞ is proportional to the global average detected rate of the cell *c*. This conclusion is important for improving the effectiveness of cooperative search and coverage.

When we implement Equation (13), there is a data overflow problem which is caused by extremely large or small value of *Q_i,c,k_*. Thus, we set a bound *Q*_max_ > 0, such that *Q_i,c,k_* ∊ [−*Q*_max_, *Q*_max_].

### 3.2. The Uncertainty Map

**Figure 3:**
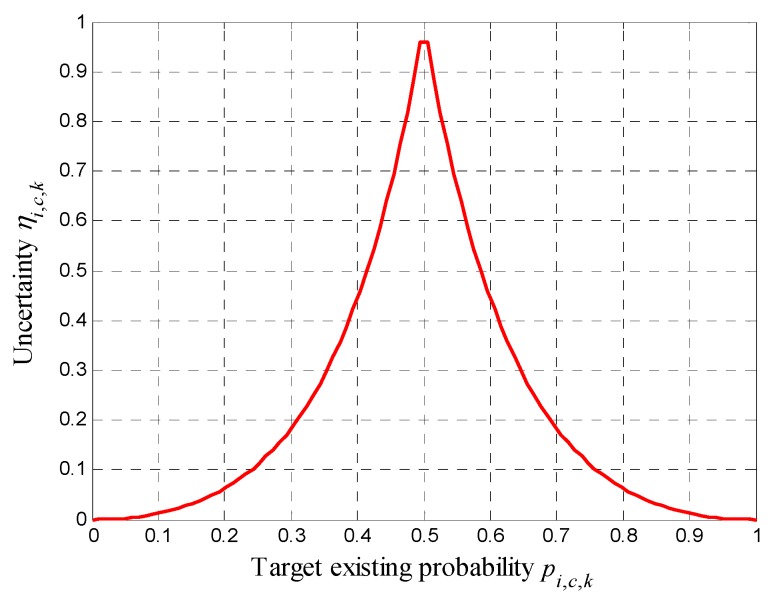
Uncertainty verse target probability in cell.

As mentioning above, if *p*_min_ < *p_i,c,k_* < *p*_max_, *A_i_* cannot confirm whether there is a target in cell *c* or not; in other words, cell *c* is uncertain for *A_i_*. In order to quantify the uncertainty of the cells in the surveillance region, we define the following uncertainty associated with cell *c*, which is a function of its target existing probability *p_i,c,k_*

(15) $$\eta_{i,c,k}=e^{-K_{\eta }\left|Q_{i,c,k}\right|}$$

in which *K_η_* > 0 is a gain parameter. |·| means absolute operator. According to Equations (8) and (15), the relationship between *η_i,c,k_* and *p_i,c,k_* is shown in Figure 3.

It is seen that when the cell *c* has *p_i,c,k_* = 0.5, it has maximal uncertainty *η_i,c,k_* = 1, which indicates that cell *c* is unknown for the UAVs completely. When cell *c* has *p_i,c,k_* = 1 or 0, it has minimal uncertainty *η_i,c,k_* = 0. In this case, the UAV are completely sure about the target present or not. In the process of cooperative search and coverage, more attention should be paid to the cells with higher uncertainties. Based on the above notations, we can define the UM as

***M***_*i*,UM_(*k*) = {*η_i,c,k_*|*c* ∊ ***Ω***} (16)

### 3.3. The Digital Pheromone Map

Theorem 3 shows the convergence speed of the TPM is proportional to the global average detected rate of the cells in the surveillance region. It means that, in order to reduce the uncertainty of the cells in the surveillance region as soon as possible, it is essential to improve the global average detected rate of the cells for the UAVs, which equates to improving the controllable revisit ability of the UAVs. For this reason, we develop a controllable revisit mechanism that is based on the characteristic of pheromone, such as “secrete, propagate and evaporation”. This mechanism can be used to control the UAVs to revisit sub-areas with large target probability or high uncertainty, and hence improve the performance of search and coverage. The digital pheromone map is defined as

***M****_i_*_,DPM_(*k*) = { *s_i_*_,*c*,*k*_|*c* ∊ ***Ω***} (17)

in which *s_i_*_,*c*,*k*_ is the pheromone strength in the cell *c* at time *k*.

Digital pheromone supports three primary operations: (i) **release**: Pheromone can be released quantitatively into the cell; (ii) **propagate**: Pheromone propagates from a cell to its neighboring cells; (iii) **evaporate**: Pheromone gradually evaporates to *zero* over time. In order to simulate these three primary operations of pheromone, the propagation factor *G*_s_ ∊ (0, 1) and the evaporation factor *E*_s_ ∊ (0, 1) are defined.

Equation (18) describes evolution of the pheromone strength in cell *c* at time *k*

(18) $$s_{i}(c,k)=(1-E_{s})\{(1-G_{s})[s_{i}(c,k-1)+k_{i}(c,k)\cdot d_{s}]+g(c,k)\}$$

in which *d_s_* is the release amount of pheromone at time *k*. *s_i_*(*c*, *k* − 1) is the pheromone strength in cell *c* at time (*k* − 1). The binary variable *k_i_*(*c*, *k*) ∊ {0, 1} is the pheromone releasing switch in cell *c* at time *k*. *g*(*c*, *k*) denotes the amount of pheromone propagated to cell *c* from its neighboring cells during the time period (*k* − 1, *k*], which can be described by the following Equation (19).

(19) $$g(c,k)=\sum_{c^{\prime }\in N(c)}\frac{G_{s}}{\left|N(c^{\prime })\right|}[s_{i}(c^{\prime },k-1)+k_{i}(c^{\prime },k)\cdot d_{s}]$$

in which ***N***(*c*) is the neighbor set of the cell *c*, the neighboring cells are denoted as *c*′ ∊ ***N***(*c*), and |***N***(*c*′)| is the number of the neighboring cells of cell *c*′. *s_i_*(*c*′, *k* − 1) is the pheromone strength in neighboring cell *c*′ at time (*k* − 1). *k_i_*(*c*′, *k*) ∊ {0, 1} is switch coefficient of the pheromone releasing in the neighboring cell *c*′ at time *k*.

The key of controllable revisit mechanism is determining the switch coefficient of the pheromone releasing in the cells of the DPM at time *k*. The switching coefficient *k_i_*(*c*, *k*) indicates whether the cell *c* autonomously releases pheromone or not. If *k_i_*(*c*, *k*) = 1, cell *c* will release the pheromone, and the released pheromone will propagate to the neighboring cells. In this way, the pheromone fields will be formed and attract the UAVs to revisit cell *c*. In order to drive the UAVs to revisit the cells that have large target probability or high uncertainty, the pheromone releasing switch of cell c should be turned on in the following two cases.

**Case 1:** If target existing probability in cell *c* satisfies the condition *p_i,c,k_* > 0.5, it indicates that the UAVs are more likely to believe that a target exists in cell *c*. However, the UAVs do not have enough evidence to support their beliefs, since *p_i,c,k_* < *p*_max_. In this case, the UAVs are required to detect cell *c* again (revisit) so as to confirm target present state of cell *c*. Thus, the pheromone releasing switch of cell *c* should be turn on (i.e., *k_i_*(*c*, *k*) = 1), in order to attract the UAVs to revisit cell *c*. Once *p_i,c,k_* ≥ *p*_max_ or *p_i,c,k_* ≤ *p*_min_, the switch should be turned off (i.e., *k_i_*(*c*, *k*) = 0) immediately, and, finally, the pheromone in cell *c* evaporates to zero over time.

**Case 2:** Assume that *τ_c_* is the last revisited time of cell *c*, *T*_0_ is a pre-defined time threshold, and *t* is current time. If (*t* − *τ_c_*) > *T*_0_, then *k_i_*(*c*, *k*) = 1, which means cell *c* has not been explored for a long time and should be revisited; otherwise, *k_i_*(*c*, *k*) = 0. Once cell *c* has revisited by a UAV, its pheromone releasing switch is turned off. After a period of time, if the condition (*t* − *τ_c_*) > *T*_0_ is satisfied again, the switch in cell *c* will be turn on again.

## 4. Distributed Path Planning Algorithm for Cooperative Search and Coverage

Optimal search and coverage paths can be designed based on the cognitive maps of the UAVs. Since the cognitive map of each UAV is incrementally updated based on its sensor observations and the shared information from other UAVs by communication, each UAV continually re-plans its path to guarantee the team of UAVs identifies maximum number of targets or gathers maximum information about environment. Therefore, multi-UAVs cooperative search and coverage problem is an on-line dynamic optimization problem. In this section, we plan optimal paths of the UAVs for cooperative search and coverage in the frame of distributed receding horizon optimizing.

### 4.1. Distributed Receding Horizon Optimizing Model for Cooperative Search and Coverage

**Figure 4:**
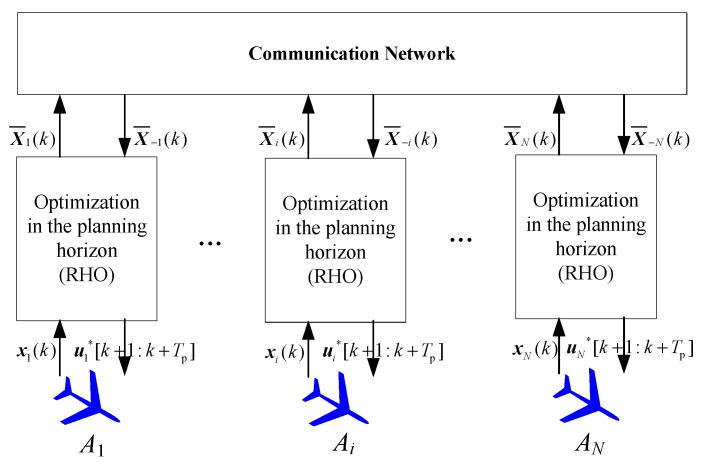
Diagram of distributed receding horizon optimizing frame for multi-UAVs search and coverage.

In the frame of distributed receding horizon optimizing as shown in Figure 4, at time *k*, *A_i_* optimizes its control inputs ${\bar{U}}_{i}(k)$ and computes its own path ${\bar{X}}_{i}(k)$ based on the received the current state ${\bar{X}}_{-i}(k)$ of its neighboring UAVs. *A_i_* computes its own path by solving the following local optimization problem

(20) $${\bar{U}}_{i}^{*}(k)=\arg\underset{{\bar{U}}_{i}(k)}{\max}J_{i}({\bar{X}}_{i}(k),{\bar{U}}_{i}(k),{\bar{X}}_{-i}(k))$$

(21) $$s.t.\{\begin{array}{c} X_{i}(k+q+1|k)=f_{i}(X_{i}(k+q|k),U_{i}(k+q|k))\\ x_{i}(k+q|k)\in \Xi \\ u_{i}(k+q|k)\in \Theta \\ q=1,2,\ldots ,T;i=1,2,\ldots ,N \end{array}$$

Define *J_i_* as the “gain” at decision time step *k*. ***f****_i_* is the UAV dynamic model. Ξ and Θ denote the feasible state set and admissible control input set of UAV, respectively. ${\bar{U}}_{i}(k)$ = {***u****_i_*(*k* + 1*|k*), …, ***u****_i_*(*k* + *T|k*)} denote the control inputs sequence of *A_i_* over the time horizon [*k* + 1:*k* + *T*], which is determined at time step *k*. According to the simplified dynamic model of UAV, the control inputs ***u*** is the choose orientation *ψ_i_* of the aircraft. ${\bar{X}}_{i}(k)$ = {***x****_i_*(*k* + 1*|k*), …, ***x****_i_*(*k* + *T|k*)} is the prediction state sequence, which is defined as the planned path of *A_i_*. ${\bar{X}}_{-i}(k)$ = {***x****_j_*(*k*)|*j* ∊ ***N****_i_*(*k*)} denote the received the current state of the neighboring UAVs of *A_i_*. Generally, in order to satisfy the collision avoidance and connectivity maintenance constraints, *A_i_* needs to obtain the future state sequence of its neighboring UAVs, denoted ${\bar{X}}_{-i}(k+q|k)$ = {***x****_j_*(*k* + 1*|k*), …, ***x****_j_*(*k* + *q|k*), …, ***x****_j_*(*k* + *T|k*)|*j* ∊ ***N****_i_*(*k*), *q* = 1, 2, …, *T*}, when *A_i_* plans its own path ${\bar{X}}_{i}^{*}(k)$ = {***x***_i_^*^(*k* + 1*|k*), …, ***x***_i_^*^ (*k* + *T|k*)}. However, due to the bandwidth limitation of the realistic wireless communication links, it is hard to receive the future state sequence of the all neighboring UAVs within one sampling time. In this paper, we adopt a feasible approach to reduce the communication between UAVs. Each UAV *A_i_* only receives the current state ${\bar{X}}_{-i}(k)$ of its neighboring UAVs. Using current state ${\bar{X}}_{-i}(k)$ and the UAV dynamic model, *A_i_* can estimate the future state sequence of the neighboring UAVs at next *T* time steps. Based on the estimate state sequence of the neighboring UAVs, *A_i_* computes its own path by solving the following local optimization problem in Equations (20) and (21). In order to guarantee the estimation accuracy and reduce the computation time, the planning horizon must be shorter.

### 4.2. Search Path Decision Process

**Figure 5:**
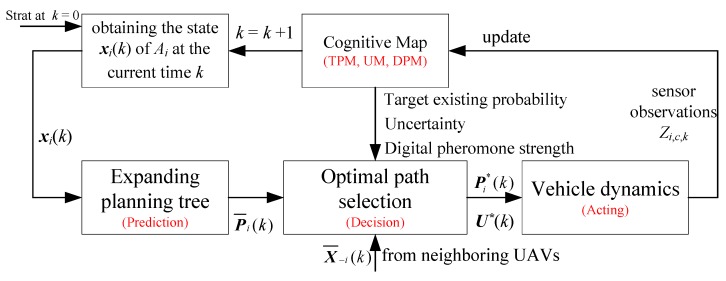
Flow diagram for single UAV search path decision strategy.

The whole process of the search path decision strategy is illustrated in Figure 5. There are three sub-stages in the whole decision process: (i) **prediction**; (ii) **decision**; and (iii) **acting**.

#### 4.2.1. Prediction Stage

In this stage, using current state ***x****_i_*(*k*) and the UAV dynamic model, each UAV *A_i_* generates its predicted state sequence, ${\bar{X}}_{i}(k)$ = {***x****_i_*(*k* + 1*|k*), …, ***x****_i_*(*k* + *q|k*), …, ***x****_i_*(*k* + *T|k*)}, at next time step, in which ***x****_i_*(*k* + *q|k*) represents the predicted state at time (*k + q*).

**Figure 6:**
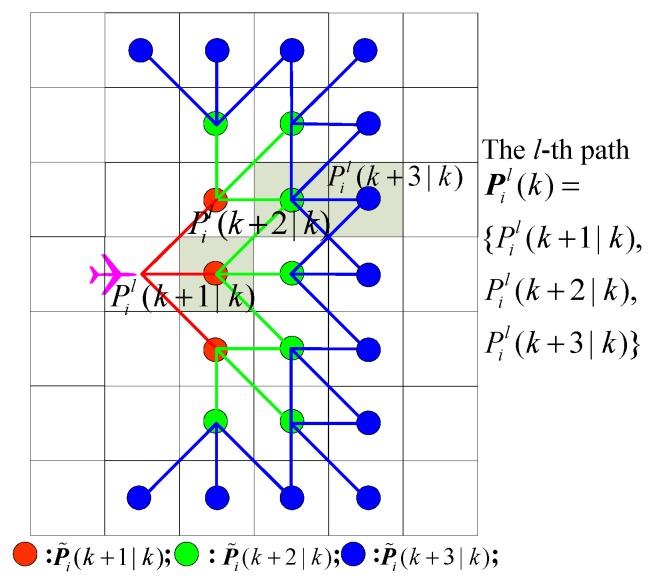
Illustration of recursive 3-step planning tree (*T* = 3).

According to the simplified UAV dynamic model, *A_i_* can only move from one cell to another neighboring cell, and has only three possible orientation choices for the next time step, i.e., turn left, go straight, and turn right, based on the current position and orientation. Therefore, the predicted state sequence ${\bar{X}}_{i}(k)$ = {***x****_i_*(*k* + 1*|k*), …, ***x****_i_*(*k* + *T|k*)} reflects the set of reachable cells from the future time step (*k* + 1) to the future time step (*k* + *q*), based on the current state ***x****_i_*(*k*) at time *k*. These reachable cells form an expanding planning tree, as shown in Figure 6. The expanding planning tree is denoted as ${\bar{P}}_{i}(k)$ = {${\tilde{P}}_{i}(k+1|k)$, …, ${\tilde{P}}_{i}(k+q|k)$, …, ${\tilde{P}}_{i}(k+T|k)$}, in which ${\tilde{P}}_{i}(k+q|k)$ is the set of the predicted reachable cells at the future time step (*k* + *q*). It is clear that the expanding planning tree contains 3*^T^* candidate paths; the *l*-th path can be denoted as ***P****_i_^l^*(*k*) = {*P_i_^l^*(*k* + 1*|k*), …, *P_i_^l^*(*k* + *q|k*), …, *P_i_^l^*(*k* + *T|k*)}, in which the waypoint (cell) *P_i_^l^*(*k* + *q|k*) ∈ ${\tilde{P}}_{i}(k+q|k)$, *q* = 1, 2, …, *T*.

#### 4.2.2. Decision Stage

In this state, in the basis of the current knowledge about the environment (such as, the target existing probability *p_i,c,k_*, the uncertainty *η_i,c,k_*, and the digital pheromone strength *s_i_*_,*c*,*k*_) available via the cognitive map, and the positions and orientations of the team of UAVs, each UAV uses a multi-objectives optimization function *J_i_* to select and update its search path. In other words, at each time step *k*, *A_i_* evaluates the value of *J_i_* associated with each path and selects the optimal path and determines the corresponding optimal control input (heading angle) sequence U*(*k*) = {ψ_i_^*^(*k* + 1*|k*), …, ψ_i_^*^(*k* + *T* − 1*|k*)}.

#### 4.2.3. Acting Stage

The first item ψ_i_^*^(*k* + 1*|k*) in the optimal decision sequence U*(*k*) is implemented to guide *A_i_* to visit the cells that waiting to be visited, and *A_i_* updates its own cognitive map into the next round of cycle.

### 4.3. Multi-Objectives of the Cooperative Search and Coverage Mission

In this section, we investigate different objectives of the search and coverage mission, which include (i) **environment exploration**; (ii) **target discovery and environment coverage**; (iii) **collision avoidance** and (iv) **connectivity maintenance**. So, we define the reward of environment exploration *J*_A_, the reward of target discovery and environment coverage *J*_B_, the cost of collision avoidance *J*_C_, and the cost of connectivity maintenance *J*_D_, respectively.

#### 4.3.1. Environment Exploration

The main objective of the mission is exploring the whole environment to decrease the uncertainty about the existence and nonexistence of the targets in the cells of the environment. In other words, the UAV should follow the path where there is maximum uncertainty in the cognitive map. Thus, if *A_i_* selects the *l*-th candidate path, ***P****_i_^l^*(*k*) = {*P_i_^l^*(*k* + 1*|k*), …, *P_i_^l^*(*k* + *q|k*), …, *P_i_^l^*(*k* + *T|k*)}, to follow, the reward of environment exploration *J*_A_ can be defined as

(22) $$J_{A}(i,l,k)=\sum_{q=1}^{T}\sum_{c\in \Phi_{i}(P_{i}^{l}(k+q|k))}\eta_{i,c,k}$$

in which ***Φ****_i_*(*c*) represents the set of cells that would be searched by *A_i_* along the path ***P****_i_^l^*(*k*).

#### 4.3.2. Target Discovery and Environment Coverage

Although the main objective of the search mission is to explore the environment to gather more information about it, one could also be interested in exploiting that information to concentrate the UAVs around the targets to capture them as soon as possible. The objective of target discovery and environment coverage is to distribute the UAVs across the environment while aggregating in more important sub-areas. These more important sub-areas refer to the cells that have high target probability (Case 1 in Section 3.3) or have not been explored for a long time (Case 2 in Section 3.3). Based on this consideration, we design the controllable revisit mechanism based on DPM in Section 3.3. Thus, if *A_i_* selects the *l*-th candidate path, ***P****_i_^l^*(*k*) = {*P_i_^l^*(*k* + 1*|k*), …, *P_i_^l^*(*k* + *q|k*), …, *P_i_^l^*(*k* + *T|k*)}, to follow, the reward of target discovery and environment coverage *J*_B_ can be defined as

(23) $$J_{B}(i,l,k)=\sum_{q=1}^{T}\sum_{c\in \Phi_{i}(P_{i}^{l}(k+q|k))}s_{i,c,k}$$

#### 4.3.3. Collision Avoidance

We use the concept of virtual rivaling force, which is borrowed from [29], to solve the collision avoidance problem. The main idea of the rivaling force mechanism is to treat the paths of other UAVs as “soft obstacles” to be avoided in path selection. The virtual rivaling force ***F****_j_*_→*i*_ exerted by *A_j_* to *A_i_* at time *k* is non-zero if the relative position and heading conditions are both hold. The relative position condition imposes the requirement that *A_j_* needs to be sufficiently close to *A_i_*. The relative heading condition means that, if *A_j_* exerts a rivaling force on *A_i_*, *A_j_* must be moving in the same or opposite direction as *A_i_*, approximately. The overall rivaling force exerted by all the other UAVs upon *A_i_* at time *k* is ***F****_i_* = ∑*_j_*_≠*i*_ ***F****_j_*_→*i*_.

**Figure 7:**
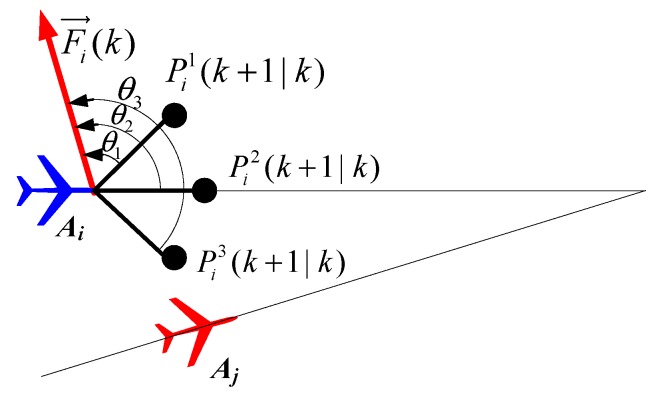
Collision avoidance based on virtual rivaling force.

A schematic diagram of multi-UAVs collision avoidance strategy using virtual rivaling force is shown in Figure 7. *P_i_*^1^(*k* + 1|*k*), *P_i_*^2^(*k* + 1|*k*), and *P_i_*^3^(*k* + 1|*k*) are three candidate waypoints; *θ*_1_, *θ*_2_, and *θ*_3_ are the angles between the direction of the virtual rivaling force ***F****_i_* and the directions in the candidate waypoints {*P_i_*^1^(*k* + 1|*k*), *P_i_*^2^(*k* + 1|*k*), and *P_i_*^3^(*k* + 1|*k*)}. The angles *θ*_1_, *θ*_2_, and *θ*_3_ satisfy 0 ≤ *θ*_1_ < *θ*_2_ < *θ*_3_ ≤ π. In order to avoid the collision between *A_i_* and *A_j_*, *A_i_* should select *P_i_*^1^(*k* + 1|*k*) as the next waypoint. Therefore, the cost of collision avoidance in waypoint *P_i_^l^*(*k* + *q*|*k*) is defined as

(24) $$J(i,P_{i}^{l}(k+q|k),k)=\exp\left(\left|F_{i}(k)|\cdot \sin(\frac{\theta (P_{i}^{l}(k+q|k))}{2}\right)\right)$$

in which |***F****_i_*| is the magnitude of the overall rivaling force ***F****_i_*. *θ*(*P_i_^l^*(*k* + *q*|*k*)) ∊ [0,π] is the angle difference between the direction of ***F****_i_* and the direction in the waypoints *P_i_^l^*(*k* + *q*|*k*). If |***F****_i_*| is small while *θ*(*P_i_^l^*(*k* + *q*|*k*)) is small, then the cost is small. Thus, if the UAV *A_i_* selects the *l*-th candidate path, ***P****_i_^l^*(*k*) = {*P_i_^l^*(*k* + 1*|k*), …, *P_i_^l^*(*k* + *q|k*), …, *P_i_^l^*(*k* + *T|k*)}, to follow, the cost of target collision avoidance *J*_C_ is

(25) $$J_{C}(i,l,k)=\sum_{q=1}^{T}J(i,P_{i}^{l}(k+q|k),k)$$

#### 4.3.4. Connectivity Maintenance

**Figure 8:**
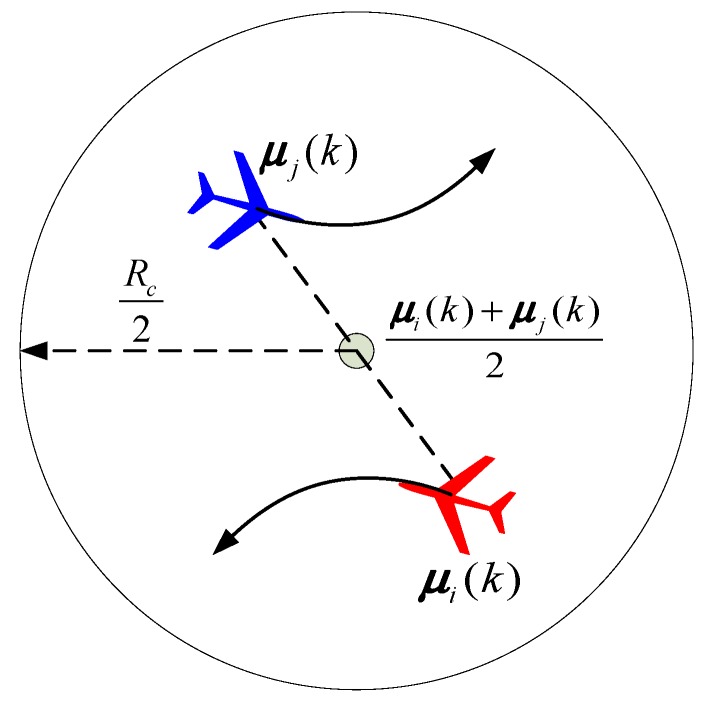
The connectivity maintenance constraint.

In order to realize the information exchange and sharing in the team of UAVs, it is usually required that the UAVs maintain a connected communication network. In this section, we present an algorithm that might be used to maintain communication connectivity. Our connectivity maintenance algorithm is based on the pairwise connectivity maintenance problem introduced in [30]. The pairwise connectivity maintenance problem is as shown in Figure 8. At time *k*, we consider two UAVs *A_i_* and *A_j_* at positions ***μ****_i_*(*k*) and ***μ****_j_*(*k*), such that ||***μ****_i_*(*k*) − ***μ****_j_*(*k*)|| ≤ *R_c_*. *R_c_* is the communication range. If *A_i_* and *A_j_* are both restricted to moving inside the disk ***ε****_i_*_,*j*,*k*_ centered at ***μ***_disk_(***μ****_i_*(*k*), ***μ****_j_*(*k*)) = 0.5[***μ****_i_*(*k*) + ***μ****_j_*(*k*)] with radius 0.5*R_c_*, then the distance between the UAVs’ positions at time (*k* + 1) is no more than *R_c_*, i.e., the communication between *A_i_* and *A_j_* is still connected at time (*k* + 1).

The disk ***ε****_i_*_,*j*,*k*_ is the connectivity constraint set, which is defined as

(26) $$\varepsilon_{i,j,k}≜\{c\in \Omega :\left\|c-\frac{\mu_{i}(k)+\mu_{j}(k)}{2}\right\|\leq \frac{R_{c}}{2}\}$$

**Figure 9:**
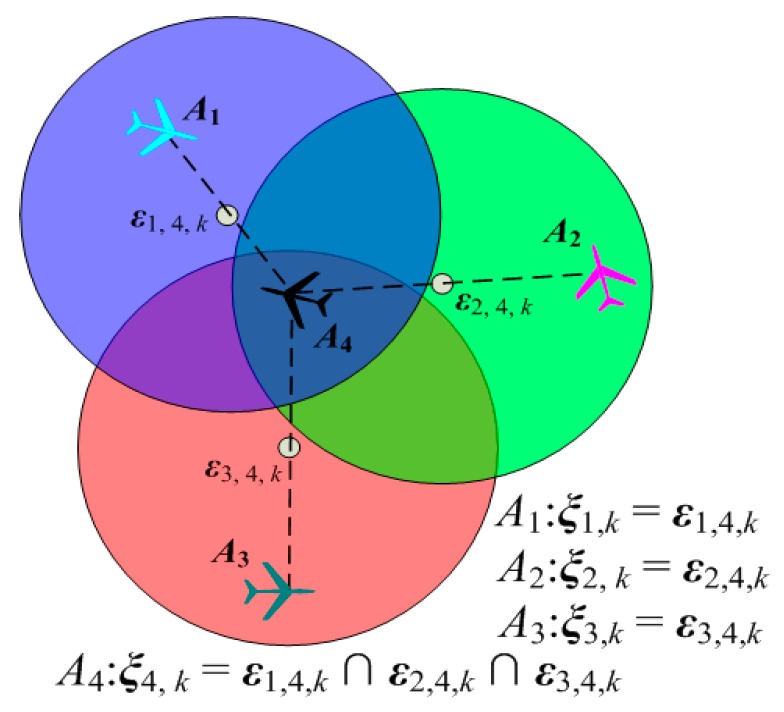
The allowable position constrained set.

Therefore, in order to maintain the network connectivity, the motion of each UAV must be restricted. Specifically, if the network is connected at time *k*, a set *ξ_i_*_,*k*_ of the allowable positions of *A_i_* need to be found. If the position of *A_i_* at time (*k* + 1) is inside the a set *ξ_i_*_,*k*_, i.e., ***μ****_i_*(*k* + 1) ∊ *ξ_i_*_,*k*_, the network is still connected at time (*k* + 1). Obviously, the allowable position constrained set ***ξ****_i_*_, *k*_ of *A_i_* is

(27) $$\xi_{i,k}≜\{\mu_{i}(k+1)\in \underset{j\in N_{i}(k)}{\cap }\varepsilon_{i,j,k}\}$$

in which ***N****_i_*(*k*) is the set of neighbors of *A_i_* at time *k*. For *A_i_*, the allowable position constrained set is determined by the intersection of all connectivity constraint sets ***ε****_i_*_,*j*,*k*_ for *j* ∊ ***N****_i_*(*k*), as shown in Figure 9.

It is clear that the network topology determines the neighbors ***N****_i_*(*k*) of *A_i_*. The number of *A_i_*’s neighbors, denoted |***N****_i_*(*k*)|, determines the size of the allowable positions set ***ξ****_i_*_,*k*_. With the increasing of |***N****_i_*(*k*)|, the size of ***ξ****_i_*_,*k*_ becomes small. This indicates that, if *A_i_* needs to maintain more links with its neighbors, then the motion space of *A_i_* will be smaller. It is not beneficial to improve the efficiency of the cooperative search and coverage. Thus, the tradeoff between the coverage enhancement and the connectivity maintenance should be considered, which aims to preserve a connected topology for the network while providing as much freedom for the UAVs as possible.

In this paper, the minimum spanning tree (MST) strategy is used to optimize the communication network topology. Based on such MST topology, only the links in the MST topology are maintained, and the redundant links are removed without violating the global connectivity condition. The Lemma 4 in [31] shows that, of all the related spanning sub-graphs ***G***_S_ applied for maintaining connectivity of network ***G***, the sub-graph ***G***_MST_ based on the MST provides the largest allowable position constrained set on average for every UAVs. The distributed algorithm for the minimum spanning tree is presented in [32].

**Figure 10:**
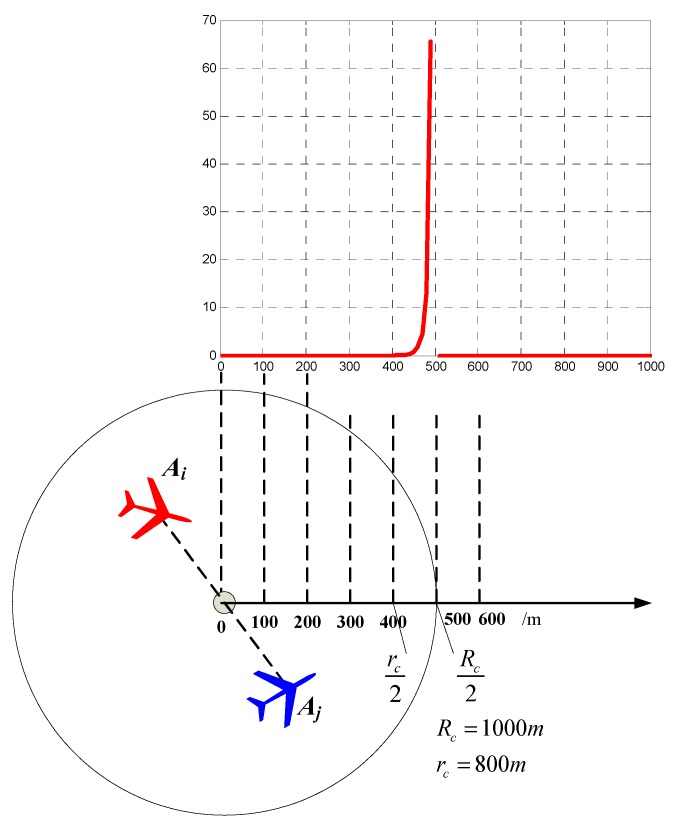
The potential field $V_{i,j,k}^{C}$ is generated to maintain the connectivity between *A_i_* and *A_j_*.

In order to ensure that the link between *A_i_* and *A_j_* is still connected at time (*k* + 1), we use the potential filed method to restrict the movement of *A_i_* inside the disk ***ε****_i_*_,*j*,*k*_. The potential field value $V_{i,j,k}^{C}(c)$ at the location of cell *c* is given by

(28) $$V_{i,j,k}^{C}(c)={\left(\min\left(0,\frac{{\left\|\mu_{c}-\mu (\varepsilon_{i,j,k})\right\|}^{2}-{\left(r_{c}/2\right)}^{2}}{{\left\|\mu_{c}-\mu (\varepsilon_{i,j,k})\right\|}^{2}-{\left(R_{c}/2\right)}^{2}}\right)\right)}^{2}$$

in which ***μ****_c_* represents the position of cell *c* in the surveillance region. ***μ***(***ε****_i_*_,*j*,*k*_) represents the center of the disk ***ε****_i_*_,*j*,*k*_. *r_c_* is a custom parameter that satisfies *r_c_* = 0.8*R_c_*. The potential field $V_{i,j,k}^{C}$ is illustrated in Figure 10. If ∆*d*(*c*, ***ε****_i_*_,*j*,*k*_) ≤ 0.5*r_c_* or ∆*d*(*c*, ***ε****_i_*_,*j*,*k*_) > 0.5*R_c_*, then $V_{i,j,k}^{C}$ = 0. If 0.5*r_c_* < ∆*d*(*c*, ***ε****_i_*_,*j*,*k*_) < 0.5*R_c_*, $V_{i,j,k}^{C}$ > 0 and $V_{i,j,k}^{C}$ sharply increase when ∆*d*(*c*, ***ε****_i_*_,*j*,*k*_) increases from 0.5*r_c_* to 0.5*R_c_*. When ∆*d*(*c*, ***ε****_i_*_,*j*,*k*_) → 0.5*R_c_*, $V_{i,j,k}^{C}$ → +∞.

Therefore, the potential field that aims to restrict the movement of *A_i_* inside the allowable position constrained set ***ξ****_i_*_, *k*_ is defined as

(29) $$V_{i,k}^{C}(c)=\sum_{j\in N_{i}(k)}V_{i,j,k}^{C}(c)$$

Thus, if the UAV *A_i_* selects the *l*-th candidate path, ***P****_i_^l^*(*k*) = {*P_i_^l^*(*k* + 1*|k*), …, *P_i_^l^*(*k* + *q|k*), …, *P_i_^l^*(*k* + *T|k*)}, to follow, the cost of connectivity maintenance *J*_D_ can be defined as

(30) $$J_{D}(i,l,k)=\sum_{q=0}^{T-1}\;V_{i,k}^{C}(P_{i}^{l}(k+q|k)$$

The total performance index function *J*(*i*, *l*, *k*) is the weighted sum of *J*_A_, *J*_B_, *J*_C_, and *J*_D_

(31) $$J(i,l,k)=\lambda_{A}J_{A}(i,l,k)+\lambda_{B}J_{B}(i,l,k)-\lambda_{C}J_{C}(i,l,k)-\lambda_{D}J_{D}(i,l,k)$$

## 5. The Simulation Validation and Results Analysis

The simulations are carried out in Microsoft Visual C++6.0 on a 2.4GHz, 2GB RAM laptop (Lenovo ThinkPad T420si, Beijing, China).

### 5.1. Effect of the Controllable Revisit Mechanism Based on Digital Pheromone

In Scenario 1, there are four UAVs performing search and coverage mission over a 2 km × 2 km rectangular region. The surveillance region is uniformly divided into 50 × 50 cells of the same size. The size of each cell is 40 m × 40 m. Three targets are scattered in the surveillance region. Table 1 and Table 2 list the initial settings of the UAVs and targets respectively. The communication range is *R*_c_ = 4000 m. Thus, the communication range is large enough to maintain the direct communication between every two vertices so that the movement of the UAVs can be not restricted by the network connectivity constraint. The sensing radius is *R*_s_ = 60 m. The detection and false alarm probabilities are *p*_d_ = 0.9 and *p*_f_ = 0.3, respectively. The time step in simulations is *T*_s_ = 0.1 s.

In Scenario 1, we compared our proposed algorithm with the method of reference [16]. The essential difference of the two methods is that the controllable revisit mechanism based on digital pheromone is not taken account by the method of reference [16]. Thus, in order to verify the controllable revisit mechanism based on digital pheromone and enhance the capacities of target capture and region coverage for the UAVs, the following two groups of experiments are carried out:

- Group A: the controllable revisit mechanism is considered (the proposed method);
- Group B: the controllable revisit mechanism is not considered (the method of reference [16]).

#### 5.1.1. Group A: With the Controllable Revisit Mechanism

**Figure 11:**
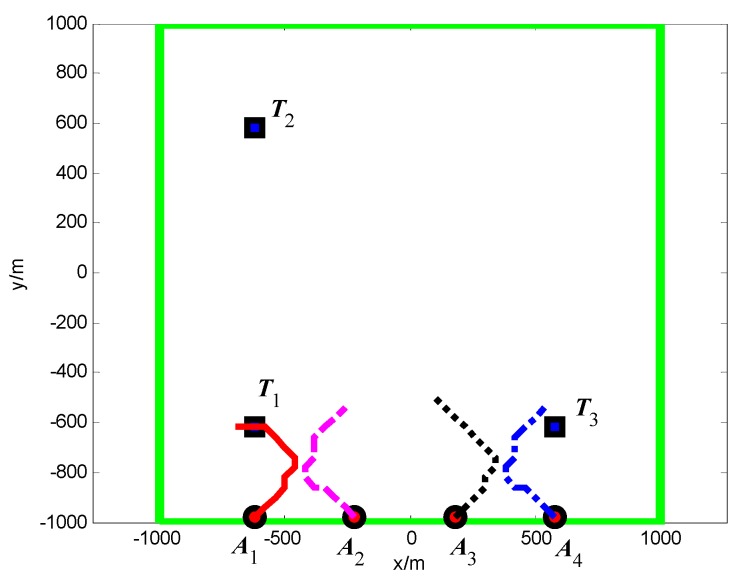
*t* = 1.4 s, *A*_1_ found *T*_1_ in cell (10, 10) (Group A in Scenario 1).

**Figure 12:**
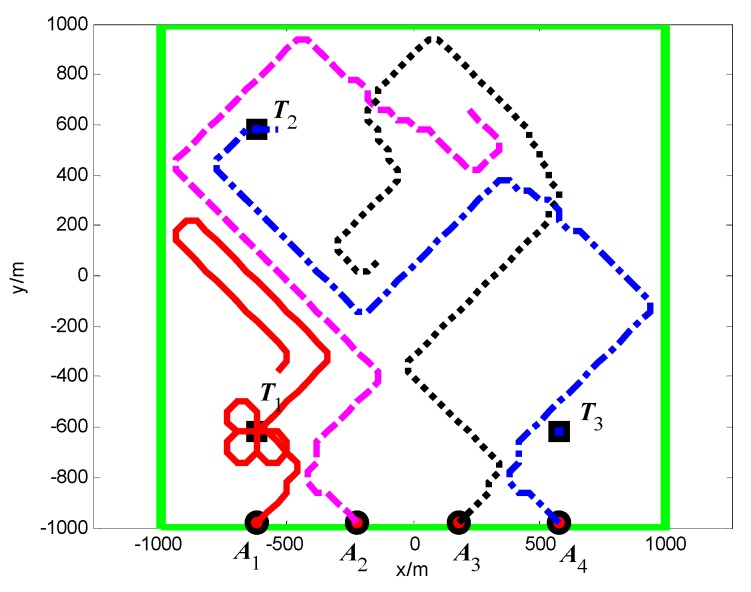
*t* = 7.7 s, *A*_4_ found *T*_2_ in cell (10, 40) (Group A in Scenario 1).

**Figure 13:**
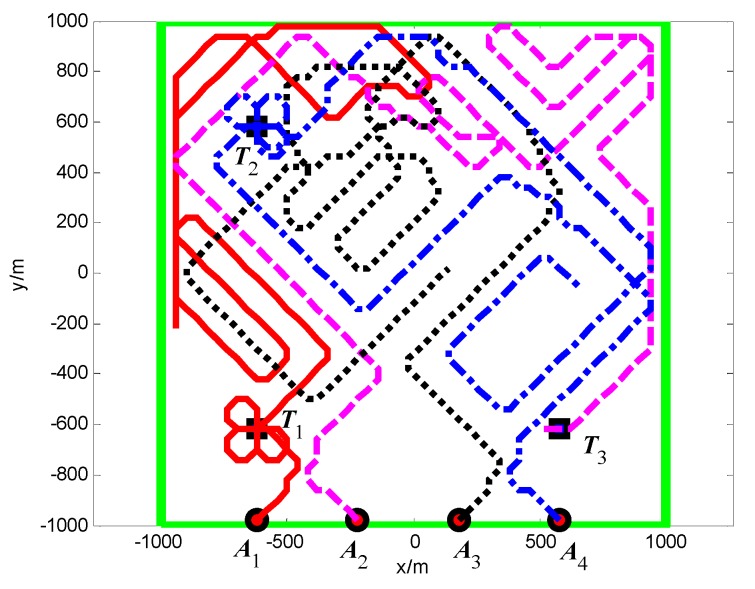
*t* = 18.2 s, *A*_2_ found *T*_3_ in cell (40, 10) (Group A in Scenario 1).

**Figure 14:**
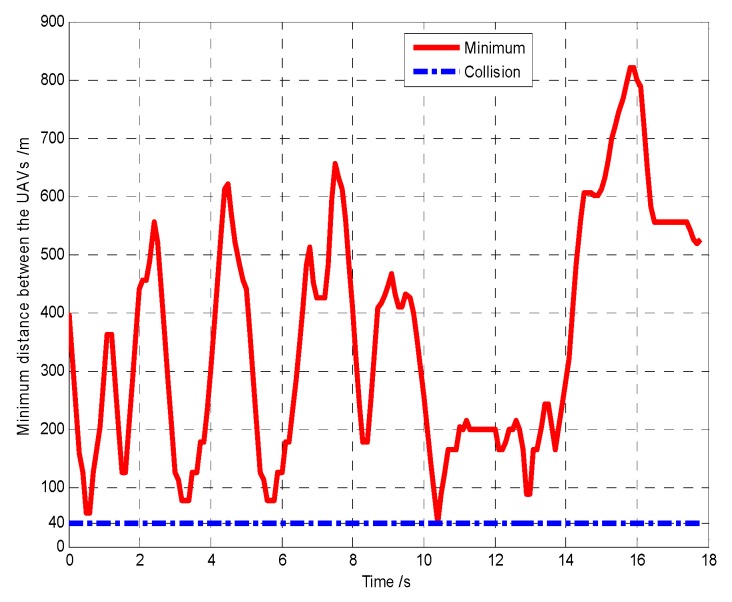
Minimum distance between the UAVs (Group A in Scenario 1).

First, when *A*_1_ arrives at the cell (10, 10) and its sensor observations is “target detection”, so the pheromone releasing switch of the cell (10, 10) in *A*_1_’s cognitive map is turned on and the cell (10, 10) releases the pheromone to attract the *A*_1_ to revisit it. At *t* = 1.4 s, as shown in Figure 11, *T*_1_ presenting in the cell (10, 10) is confirmed by *A*_1_. Then, *A*_4_ arrives at the cell (10, 40), and its sensor observation is “target detection”, so the cell (10, 40) releases the pheromone to attract the *A*_4_ to revisit it. At *t* = 7.7 s, as shown in Figure 12, *T*_2_ presenting in the cell (10, 40) is confirmed by *A*_4_. At last, at *t* = 18.2 s, *T*_3_ in the cell (40, 10) is confirmed by *A*_2_, as shown in Figure 13. Figure 14 shows the minimum distance between the UAVs. The collision distance is the length of the square cell, which is 40 m. The minimum distance is never lower than the collision distance; it means that any two UAVs are always not in the same cell; thus, the collision avoidance is guaranteed.

#### 5.1.2. Group B: Without the Controllable Revisit Mechanism

**Figure 15:**
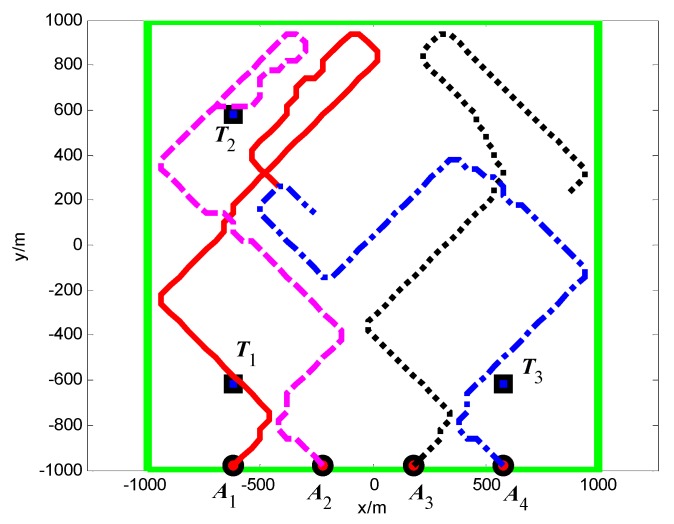
*t* = 6.9 s, *A*_2_ found *T*_2_ in cell (10, 40) (Group B in Scenario 1).

**Figure 16:**
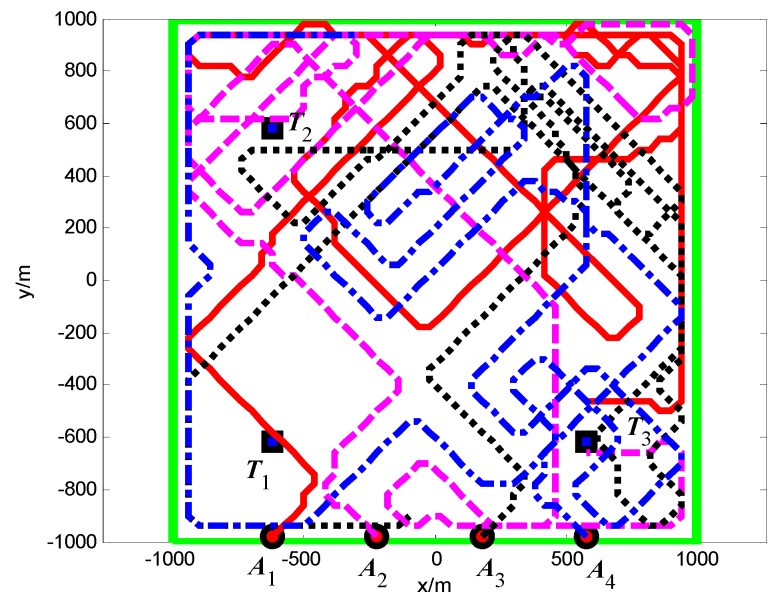
*t* = 30.2 s, *A*_2_ found *T*_3_ in cell (40, 10) (Group B in Scenario 1).

**Figure 17:**
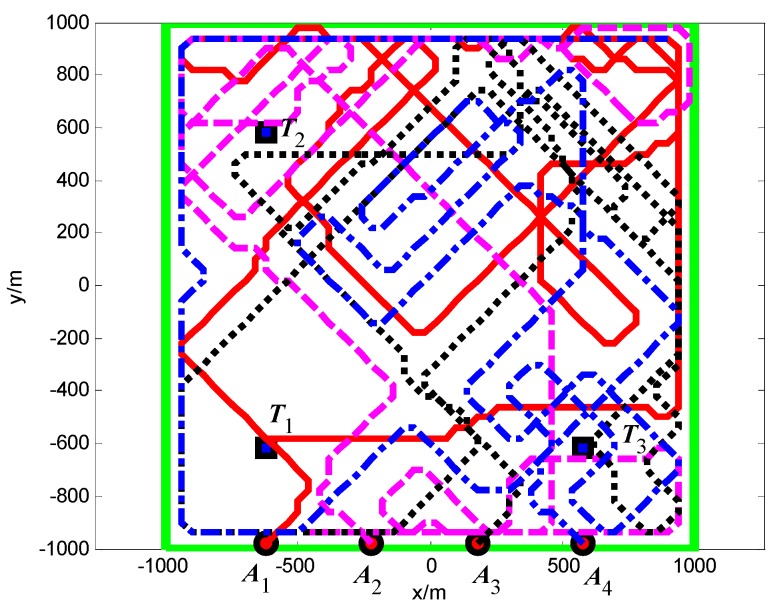
*t* = 33.2 s, *A*_1_ found *T*_1_ in cell (10, 10) (Group B in Scenario 1).

**Figure 18:**
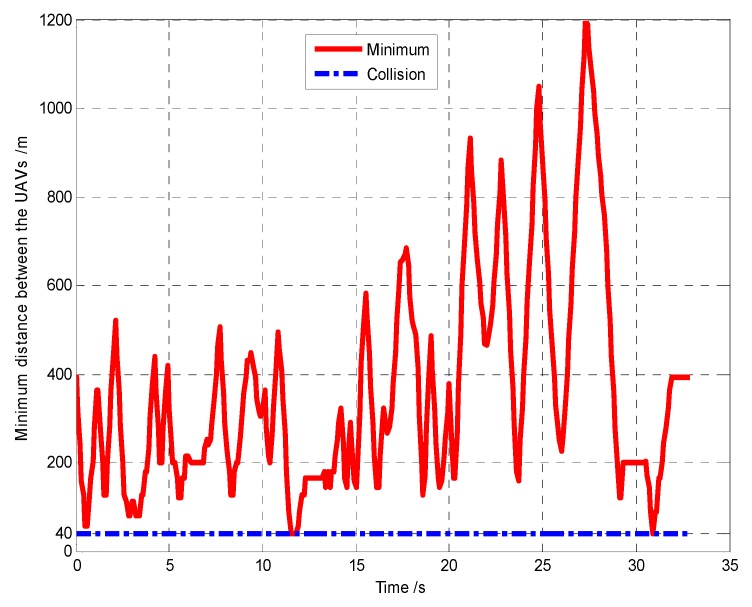
Minimum distance between the UAVs (Group B in Scenario 1).

The snapshots of finding the targets *T*_2_, *T*_3_, *T*_1_ are shown in Figure 15, Figure 16 and Figure 17, respectively. From these snapshots, we can conclude that, due to lacking the controllable revisit mechanism, the capabilities of the UAVs for target capture in Group B are lower than Group A. Specifically, as shown in Figure 15, although *A*_1_ travels through the cell (10, 10) at the beginning of the search process, *A*_1_ does not revisit the cell (10, 10) to confirm whether there is a target in this cell or not, due to lacking the controllable revisit mechanism. Therefore, the target *T*_1_ is not captured (found and confirmed) early enough and is confirmed by *A*_1_ until *t* = 33.2 s, as shown in Figure 17. Figure 18 shows the minimum distance between the UAVs in Group B. It can be seen that two UAVs are never in the same cell; thus, the collision avoidance is guaranteed.

From the time when the targets are confirmed by the UAVs in Groups A and B, it can be concluded that the controllable revisit mechanism can concentrate the UAVs around the targets to capture them as soon as possible, and enhance the capacity of target capture for the UAVs.

**Figure 19:**
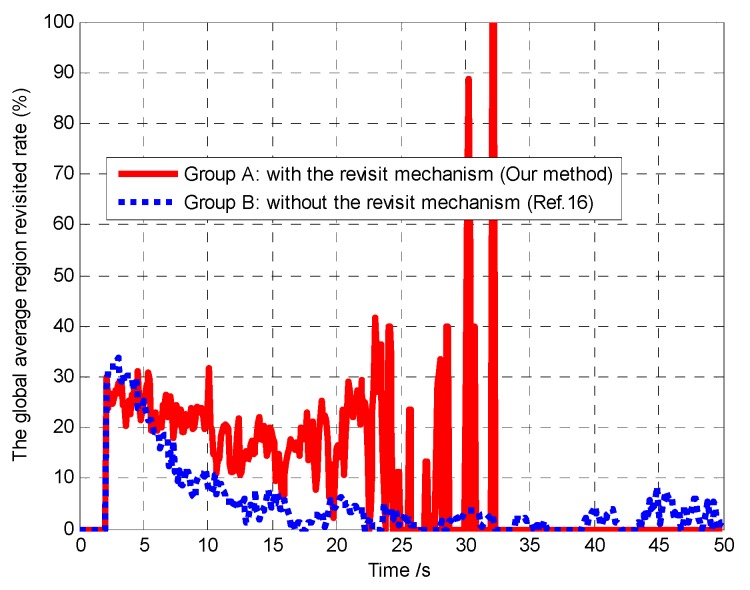
Comparison of the global average revisited rate (Scenario 1).

**Figure 20:**
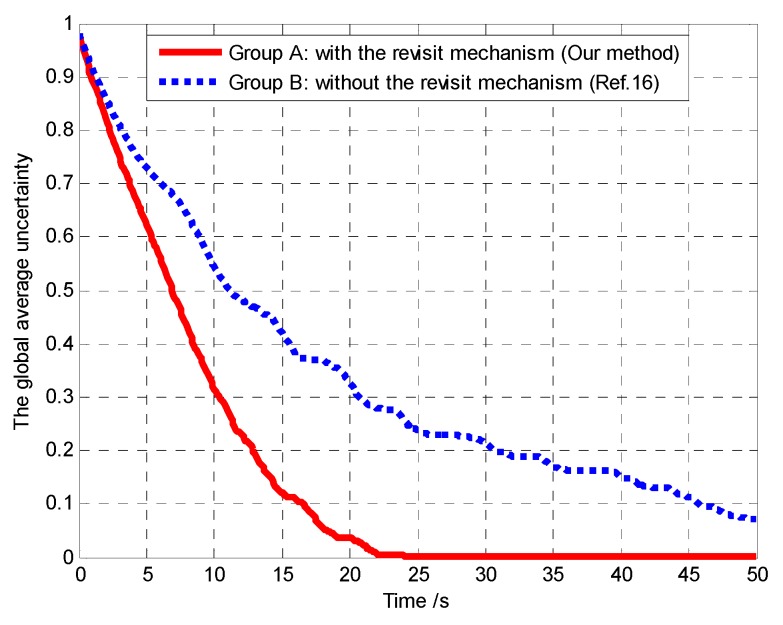
Comparison of the global average uncertainty (Scenario 1).

Furthermore, in order to verify that the controllable revisit mechanism can enhance the region coverage capacity of the UAVs and then improve the performance of mission operation, we analyze and compare the global average region revisited rate and the global average uncertainty in Groups A and B, as shown in Figure 19 and Figure 20, respectively.

First, we defined the following global average region revisited rate (“global” means it is averaged over the *N* UAVs and (*L_x_* × *L_y_*) cells) to evaluate the region coverage capacity of the UAVs.

(32) $${\bar{\sigma }}_{k}=\left(\frac{1}{N}\sum_{i=1}^{N}\sigma_{i,k}\right)\times 100\%=\left(\frac{1}{N}\sum_{i=1}^{N}\frac{\vartheta_{i,k}}{\varepsilon_{i,k}}\right)\times 100\%$$

in which ${\bar{\sigma }}_{k}$ is the global average region revisited rate at time *k*. *σ_i_*_,*k*_ indicates the region revisited rate of *A_i_* at time *k*. *ϑ_i_*_,*k*_ indicates the number of the cells that are being revisited by *A_i_* at time *k*. *ε_i_*_,*k*_ indicates the number of the cells that need to be revisited at time *k* in the cognitive map of *A_i_*. Generally, *ϑ_i_*_,*k*_ ≤ *ε_i_*_,*k*_, and if *ε_i_*_,*k*_ = 0, we set *σ_i_*_,*k*_ = 0.

Then, we defined the following global average uncertainty to evaluate the performance of mission operation.

(33) $${\bar{\eta }}_{k}=\frac{1}{N(L_{x}\times L_{y})}\sum_{i=1}^{N}\sum_{c\in \Omega }\eta_{i,c,k}$$

It can be seen from Figure 19 that the global average region revisited rate of the cells in Group A is higher than that in Group B, due to considering the controllable revisit mechanism in Group A and lacking the controllable revisit mechanism in Group B. In Group A, after about 35 s, the target existence status of most of the cells has been confirmed, so that the average revisited rate remains substantially zero after about 35 s. However, at some moments (e.g., *t* = 32.2 s), the average revisited rate is approximately 100%. This is because the number of cells currently being revisited is approximately equal to the number of cells that need to be revisited at these moments. Thus, we can conclude that the controllable revisit mechanism can enhance the region coverage capacity of the UAVs.

It can be seen from Figure 20 that compared with Group B, the global average uncertainty in Group A decreases to 0 more quickly. In the controllable revisit mechanism, we design the digital pheromone as the “guidance information”, which is used to concentrate the UAVs to revisit sub mission area with high target probability or maximum uncertainty. In this way, the global average detected rate of the cell increases accordingly. Therefore, according to Theorem 3, in Group A the convergence speed of the cognitive maps in the UAVs is higher than in Group B, which means that our method has better mission operation performance than the method in reference [16].

### 5.2. Effect of Different Connectivity Maintenance Control Strategies

In Scenario 2, we set limited communication radius *R*_c_ = 1000 m and compared our proposed algorithm with the method of reference [26]. The essential difference of the two methods is that, in our method, the MST topology strategy is used to optimize the communication network topology, and only the communication links in the MST topology are maintained without violating the global connectivity condition. In the method of reference [26], the communication network topology is full connected, which means that the links between each vehicle must be maintained during the mission process. Thus, in order to test the influence of different connectivity maintenance control strategies on the performance of mission operation, the following two groups of experiments are carried out:

- Group A: the MST topology strategy (the proposed method);
- Group B: the full connected topology (the method of reference [26]).

#### 5.2.1. Group A: The Minimum Spanning Tree Topology

**Figure 21:**
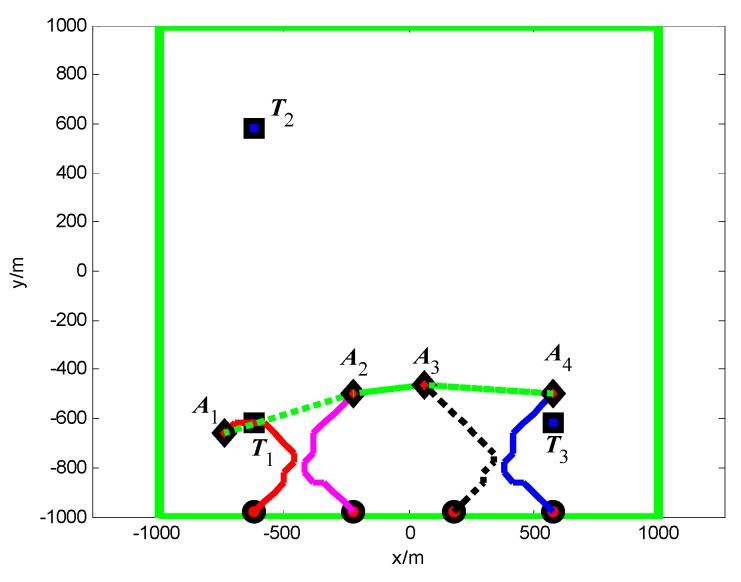
*t* = 1.4 s, *A*_1_ found *T*_1_ in cell (10, 10) (Group A in Scenario 2).

**Figure 22:**
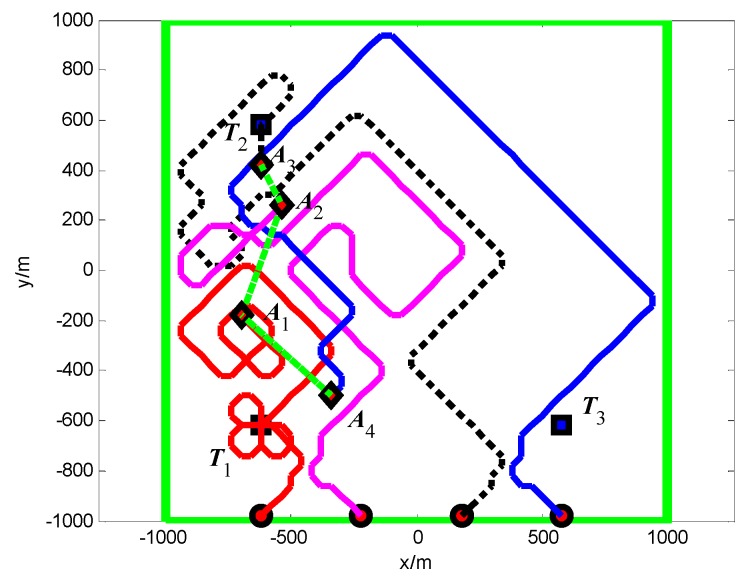
*t* = 8.9 s, *A*_3_ found *T*_2_ in cell (10, 40) (Group A in Scenario 2).

**Figure 23:**
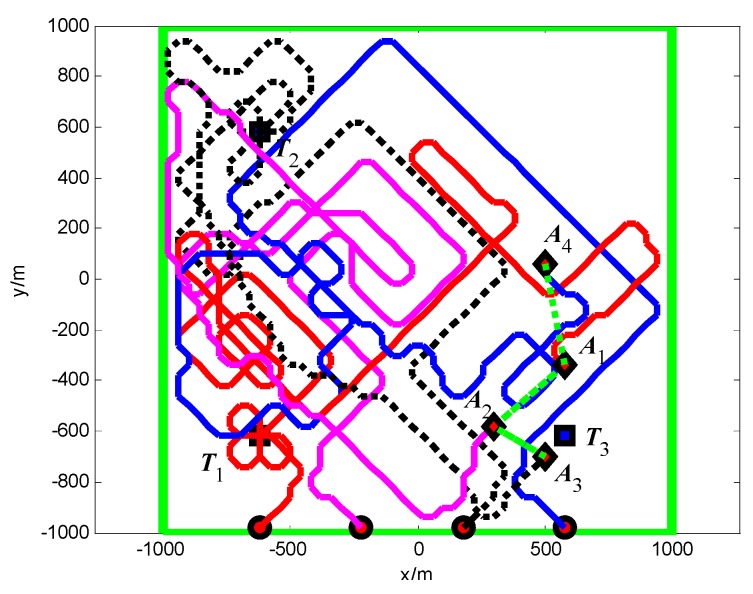
*t* = 19.4 s, *A*_3_ found *T*_3_ in cell (40, 10) (Group A in Scenario 2).

**Figure 24:**
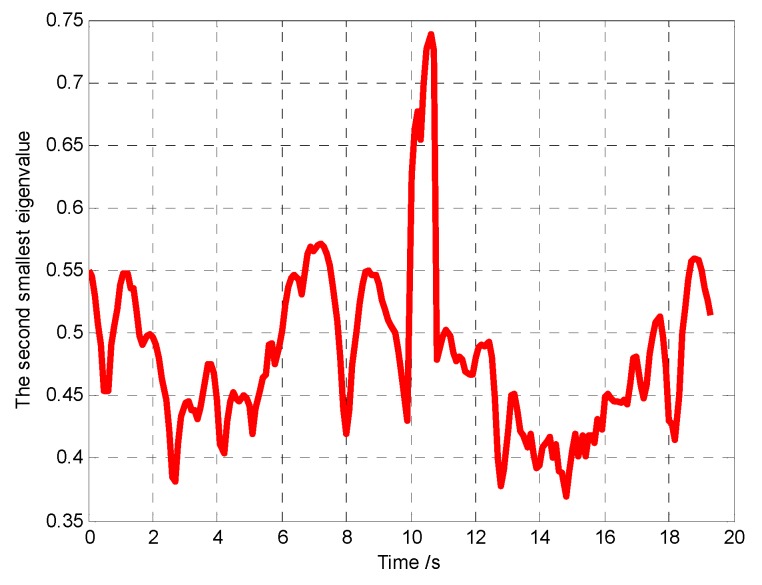
The second smallest eigenvalue (Group A in Scenario 2).

The snapshots, Figure 21, Figure 22 and Figure 23, respectively, show the search paths and communication topology of the whole UAVs when the targets *T*_1_, *T*_2_, *T*_3_ are found in Group A. The communication topology of the UAVs is denoted by the green dashed lines in these snapshots; it can be seen that the minimum spanning tree is used to optimize the topology of the communication network during the search process, and only the communication links in the MST topology are maintained. The second smallest eigenvalue of the Laplacian matrix of the communication topology is illustrated in Figure 24. It can be seen that second smallest eigenvalue is always larger than zero, so the network connectivity is maintained during the mission process.

#### 5.2.2. Group B: The Full Connected Topology

**Figure 25:**
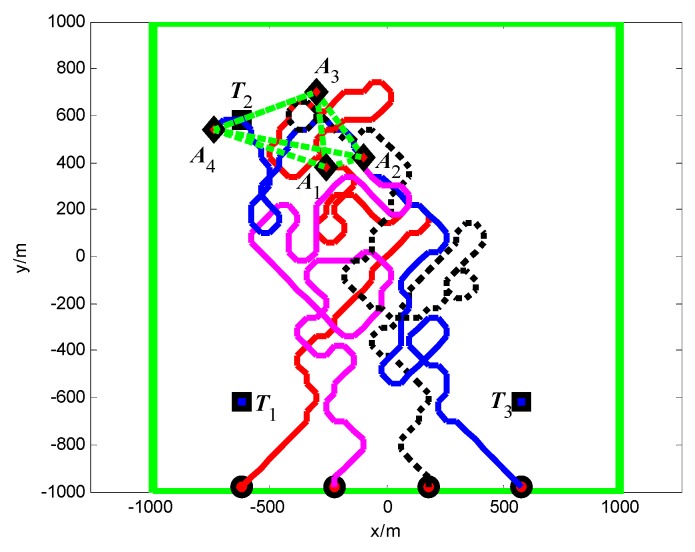
*t* = 9.2 s, *A*_4_ found *T*_2_ in cell (10, 40) (Group B in Scenario 2).

**Figure 26:**
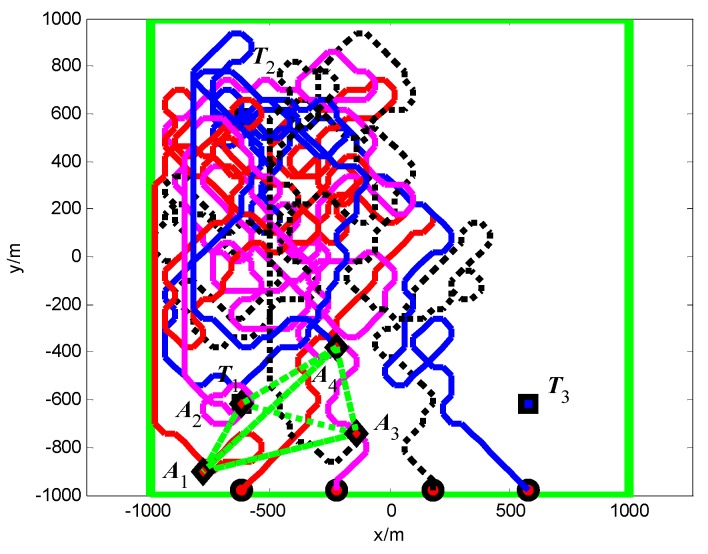
*t* = 27.9, *A*_2_ found *T*_1_ in cell (10, 10) (Group B in Scenario 2).

**Figure 27:**
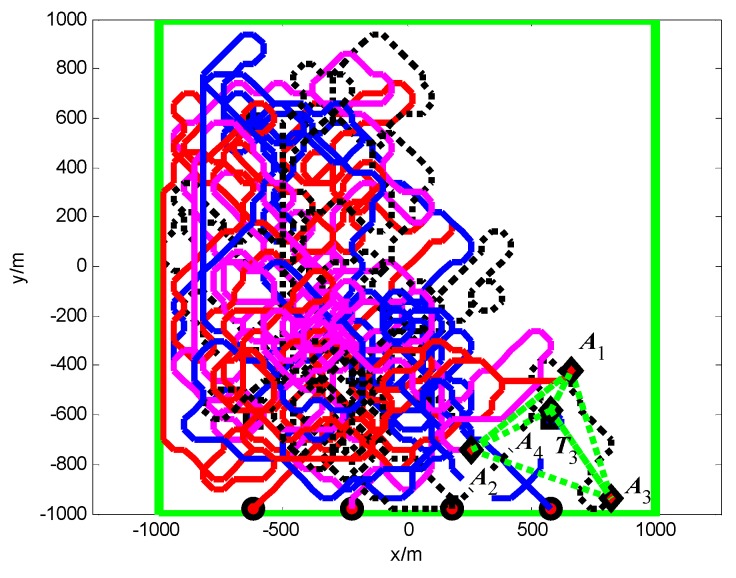
*t* = 56.0 s, *A*_4_ found *T*_3_ in cell (40, 10) (Group B in Scenario 2).

**Figure 28:**
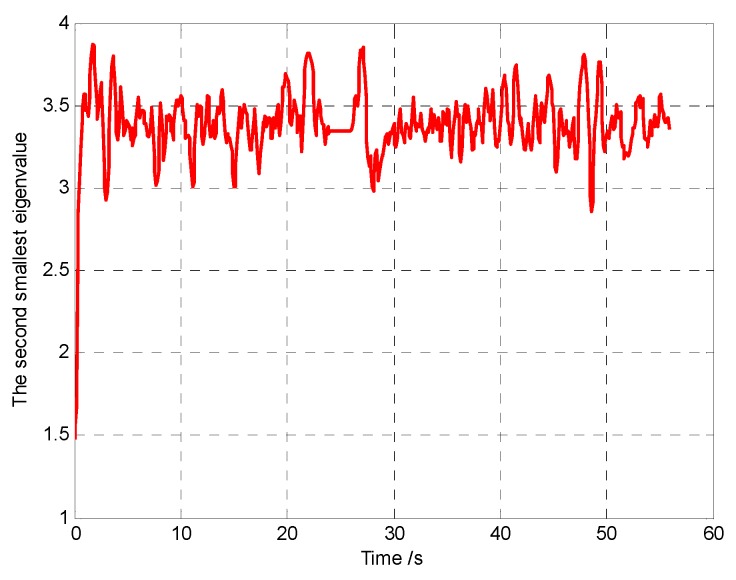
The second smallest eigenvalue (Group B in Scenario 2).

The snapshots, Figure 25, Figure 26 and Figure 27, respectively, show the search paths and communication topology of the whole UAVs when the targets *T*_2_, *T*_1_, *T*_3_ are found in Group B. As shown in Figure 27, in order to maintain a fully connected communication topology, the UAVs are concentrated around the left half plane in the surveillance region. This causes the right half plane in the surveillance region that is not explored by the UAVs. The second smallest eigenvalue of the Laplacian matrix of the network topology is illustrated in Figure 28. It can be seen that the network connectivity is maintained during the mission process.

**Figure 29:**
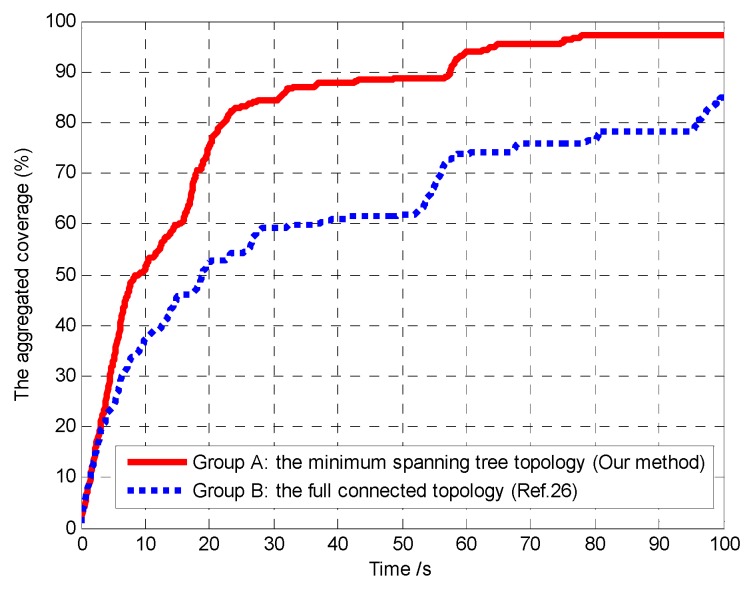
Comparison of the aggregated coverage (Scenario 2).

**Figure 30:**
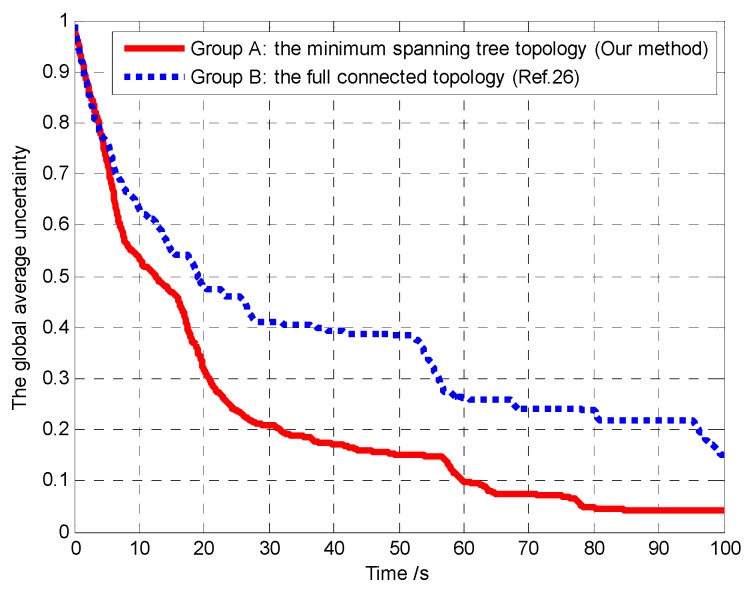
Comparison of the global average uncertainty (Scenario 2).

Furthermore, in order to verify the connectivity maintenance scheme based on the MST topology optimization and efficaciously balance the coverage enhancement and the connectivity maintenance, and then improve the performance of mission operation, we analyze and compare the aggregated coverage of the whole surveillance region and the global average uncertainty in Groups A and B, as shown in Figure 29 and Figure 30, respectively.

In Scenario 2, we defined the following aggregated coverage of the surveillance region for the UAVs to evaluate the region coverage capacity of the UAVs.

(34) $$\varpi_{k}=\frac{\omega_{k}}{L_{x}\cdot L_{y}}\times 100\%$$

in which $\varpi_{k}$ denotes the aggregated coverage at time *k*. *ω_k_* indicates the aggregated number of the cells that have been searched at least once by at least one UAV up to time *k*.

Then, we also use the global average uncertainty, which is defined in Equation (33), to evaluate the performance of mission operation.

It can be seen that, in Group A, the aggregated coverage is higher than Group B (Figure 29), and the convergence speed of the cognitive maps in the whole UAVs is higher than Group B (Figure 30), which means that our method has better mission operation performance than the method in reference [26]. The reason for these results is that the connectivity maintenance scheme based on the MST communication topology optimization relaxes the communication constraints and gives more freedom for the UAVs to search the more areas. However in Group B the UAVs tend to maintain all the communication links with the other UAVs rather than exploring more areas. Hence, the connectivity maintenance scheme based on the MST communication topology optimization can efficaciously balance the coverage enhancement and the connectivity maintenance, which aim to maintain a connected topology for the network while minimizing the movement constraints on the UAVs.

### 5.3. Effect of Varying Number of UAVs

In Scenario 3, we use different number of UAVs to test its influence on the average mission complete time (AMCT). In Monte-Carlo simulations, the number of targets is *M* = 3, while the number of UAVs is *N* = 5, 6 and 7; thus, the simulations can be divided into 3 groups of experiments. The number of experiments was 100 in each group. We need to calculate the AMCT of each group of experiments. The mission completion time is defined as the required mission execution time when the global average uncertainty $\bar{\eta }$ ≤ 0.01. For each experiment, the initial positions and the initial heading angles of the UAVs, and the initial positions of targets, are randomly generated. The communication range is *R*_c_ = 4000 m. Thus, the communication range is large enough to maintain direct communication between each vertice so that the movement of the UAVs can be not restricted by the network connectivity constraint. The sensing radius is *R*_s_ = 60 m. The detection and false alarm probabilities are *p*_d_ = 0.9 and *p*_f_ = 0.3, respectively.

**Figure 31:**
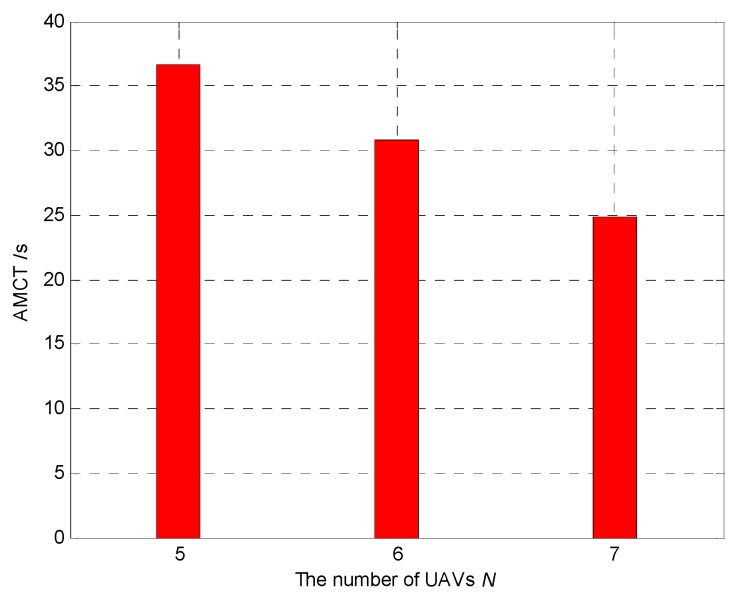
Effect of varying number of UAVs on the AMCT (Scenario 3).

Figure 31 shows the AMCT for different numbers of UAVs, and we can summarize that the larger the number of UAVs, the smaller the AMCT. This is because if the number of UAVs is larger, more cells in the surveillance region are detected by the UAVs, and hence the global average detection rate of the cell will be higher. According to Theorem 3, the convergence speed of the cognitive maps in the UAVs is higher, so the AMCT is smaller.

### 5.4. Effect of Different Sensing Radius

In Scenario 4, we set different values of sensing radius to test its influence on the AMCT. In Monte-Carlo simulations, the sensing radius is 20 m, 60 m, 100 m if the number of UAVs is kept as *N* = 5 and the number of targets is kept as *M* = 3. The communication range is *R*_c_ = 4000 m. The detection and false alarm probabilities are *p*_d_ = 0.9 and *p*_f_ = 0.3, respectively. In Scenario 4, the mission completion time is defined as the required mission execution time when the global average uncertainty $\bar{\eta }$ ≤ 0.1. For each experiment, the initial positions and the initial heading angles of the UAVs, and the initial positions of targets, are randomly generated.

**Figure 32:**
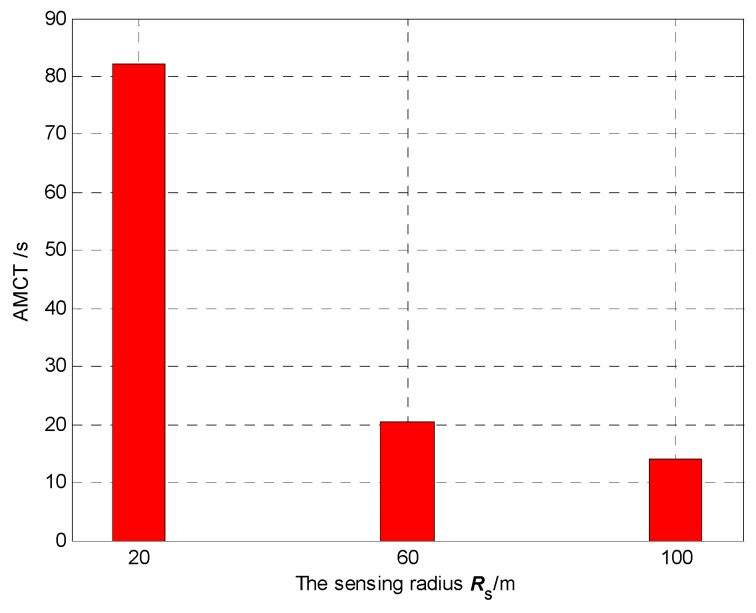
Effect of varying sensing radius on the AMCT (Scenario 4).

Figure 32 shows the AMCT for different values of sensing radius, and we can summarize that the larger the sensing radius, the smaller the AMCT. This is because if the sensing radius is larger, more cells in the surveillance region are detected by the UAVs, and hence the global average detected rate of the cell will be higher. According to Theorem 3, the convergence speed of the cognitive maps in the UAVs is higher, so the AMCT is smaller.

### 5.5. Effect of Detection and False Alarm Probabilities

In Scenario 5, we set different detection probabilities and different false alarm probabilities to test their influence on the AMCT. In Monte-Carlo simulations, the detection probability *p*_d_ is 0.6, 0.7 and 0.9, while the false alarm probability *p*_f_ is 0.2, 0.3 and 0.4. The number of targets is *M* = 3. The number of UAVs is *N* = 5. The communication range is *R*_c_ = 4000 m. The sensing radius is *R*_s_ = 60 m. In Scenario 5, the mission completion time is defined as the required mission execution time when the global average uncertainty $\bar{\eta }$ ≤ 0.01. For each experiment, the initial positions and the initial heading angles of the UAVs, and the initial positions of targets, are randomly generated.

**Figure 33:**
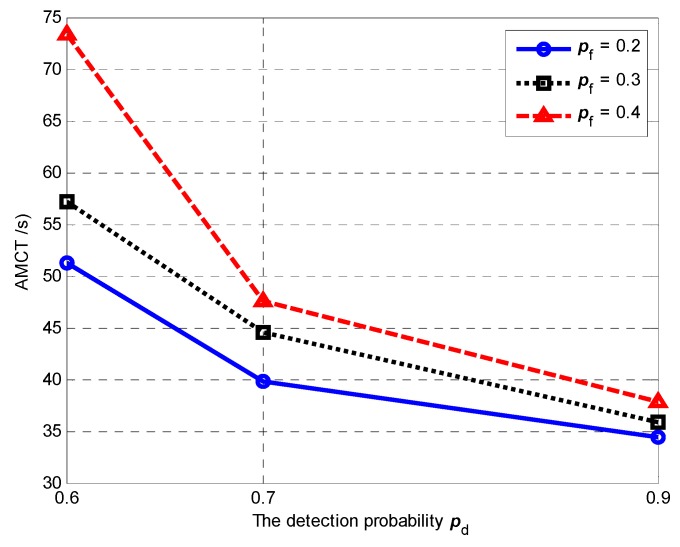
Effect of various detection and false alarm probabilities on the AMCT (Scenario 5).

Figure 33 shows the AMCT for different detection probabilities and different false alarm probabilities, and we can summarize that, for a given detection probability *p*_d_, the smaller the false alarm probability *p*_f_, the smaller the AMCT. For a given false alarm probability *p*_f_, the larger the detection probability *p*_d_ and the smaller the AMCT. According to Theorem 2, the better the performance of the sensor is, such as the larger the detection probability *p*_d_ and the smaller the false alarm probability *p*_f_, the minimum number of observations that require $m_{\mathrm{avg}}^{+}$ or $m_{\mathrm{avg}}^{-}$ is smaller. In other words, the target existence status in each cell can be confirmed by fewer searches, and hence the AMCT is smaller.

### 5.6. Effect of Different Communication Range

In Scenario 6, we set different communication ranges to test their influence on the AMCT. In Monte-Carlo simulations, the communication range *R*_c_ is 800 m, 1000 m, 1200 m, 1500 m and 1800 m, and keep the number of UAVs is *N* = 4; the number of targets as *M* = 3. The sensing radius is *R*_s_ = 60 m. The detection and false alarm probabilities are *p*_d_ = 0.9 and *p*_f_ = 0.3, respectively. For each experiment, the initial positions and the initial heading angles of the UAVs, and the initial positions of targets, are randomly generated. In Scenario 6, the mission completion time is defined as the required mission execution time when the global average uncertainty $\bar{\eta }$ ≤ 0.1.

**Figure 34:**
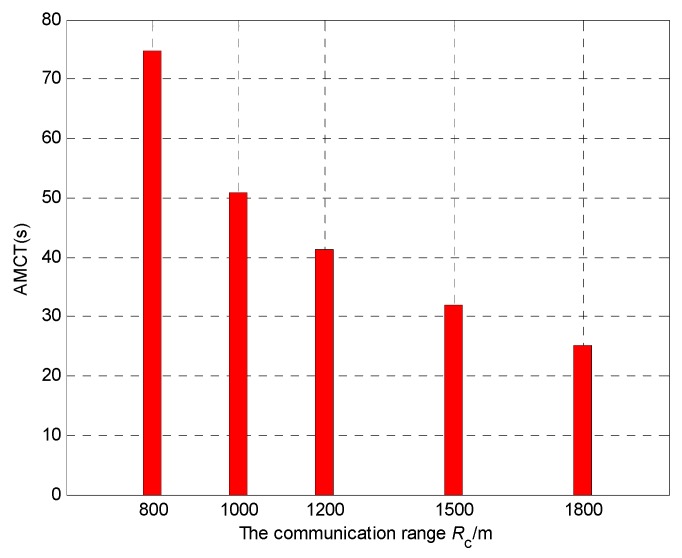
Effect of various communication ranges on the AMCT (Scenario 6).

Figure 34 shows the AMCT for different communication ranges, and we can summarize that the larger the communication range, the smaller the AMCT. This is because if the communication range is larger, the size of the connectivity constraint set ***ε****_i_*_,*j*,*k*_ is larger, and then the size of the allowable positions set ***ξ****_i_*_,*k*_ is larger, which gives more freedom for the UAVs without violating the network connectivity constraint. In this case, the UAVs can search more areas, and which leads to the higher average detected rate of the cell. According to Theorem 3, the convergence speed of the cognitive maps in the UAVs is higher, so the AMCT is smaller. However, it is worth noting that when the communication range is large enough, the AMCT is essentially unchanged. This is because if the communication range is large enough to maintain the direct communication links between every two vertices, the movements of the UAVs in the surveillance region are not bound by the communication range constraint. This means that the communication between UAVs can be seen as perfect, so that the influence of the communication range can be ignored.

It is also worth noting that, in Scenario 1, 3, 4 and 5, the communication range is large enough to maintain the direct communication between every two vertices so that each UAV can exchange the target probability maps with all the other UAVs. Thus, there is no deviation between each two individual target probability maps. However, in Scenario 6, due to limited communication range, there exits the deviation between each two individual target probability maps. Hence, in Scenario 6, on one hand, we use the global average uncertainty, which is defined in Equation (33), to evaluate the convergence performance of the target probability maps in the UAVs. On the other hand, the following global average uncertainty deviation is defined to evaluate the consensus performance of the UAVs, which implement the distributed update and fusion scheme in Equation (13) for map merging.

(35) $$\Delta {\bar{\eta }}_{k}=\frac{1}{N(L_{x}\times L_{y})}\sum_{i=1}^{N}\sum_{c\in \Omega }\left(\left|\eta_{i,c,k}-{\bar{\eta }}_{k}\right|\right)$$

**Figure 35:**
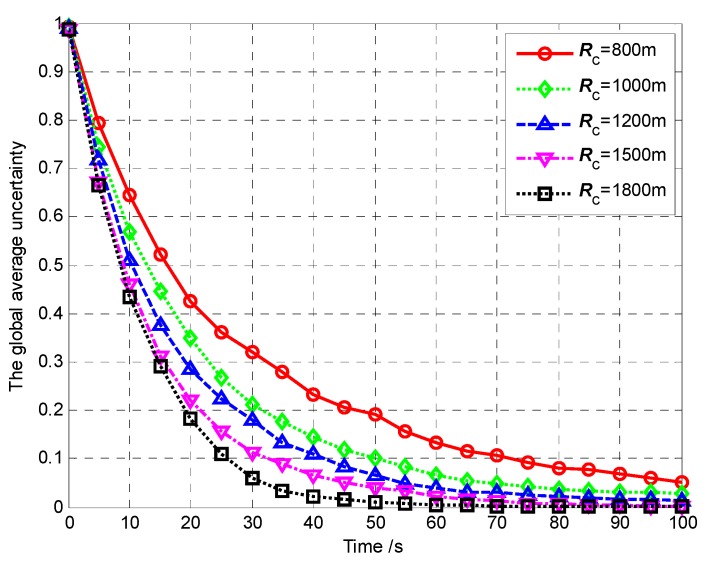
The global average uncertainty for the different communication ranges (Scenario 6).

**Figure 36:**
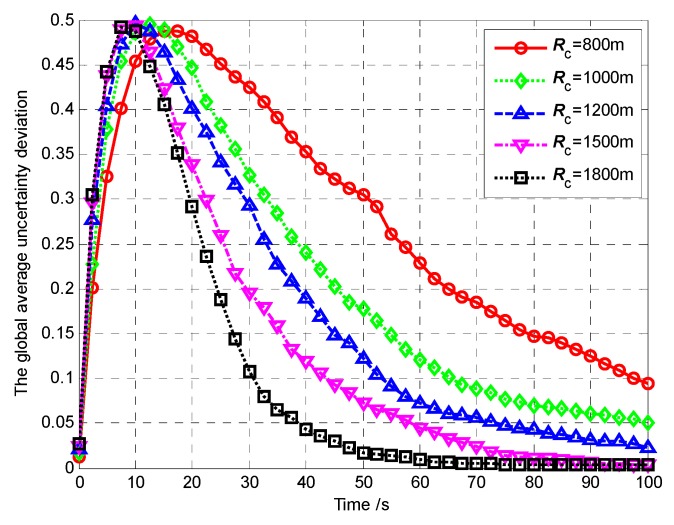
The global average uncertainty deviation for the different communication ranges (Scenario 6).

Figure 35 and Figure 36 show the global average uncertainty and the global average uncertainty deviation for the different communication ranges, respectively.

From Figure 35, it can be seen that the larger communication range, the faster the global average uncertainty decreases to 0. This is because the larger communication range gives more freedom for the UAVs without violating the network connectivity constraint. In this case, the UAVs can search more areas, which leads to the higher average detected rate of the cells. According to Theorem 3, the map convergence speed of the whole UAVs is higher, which means the performance of mission operation is better (this conclusion is also confirmed in Figure 34).

From Figure 36, we can summarize that the larger the communication range, the faster the global average deviation of the uncertainties decreases to 0, which means the consensus performance is better. This is because, the larger the communication range, the more communication neighbors of each UAV *A_i_*. It means that more UAVs share their observation results with *A_i_*. It is beneficial to eliminate deviations between the individual probability maps of the UAVs.

### 5.7. Comparison of the Two Map Update Methods

To evaluate the effectiveness of our proposed distributed update and fusion scheme, two groups of experiments are carried out in Scenario 7.

- Group A: **uncooperative** **map update**. Each UAV only updates its own TPM according to its sensor observations.
- Group B: **cooperative map merging**. Each UAV first updates its own TPM according to its sensor observations, and then transmits the updated map to its neighbors for map fusion using our proposed update and fusion scheme in Equation (3).

**Figure 37:**
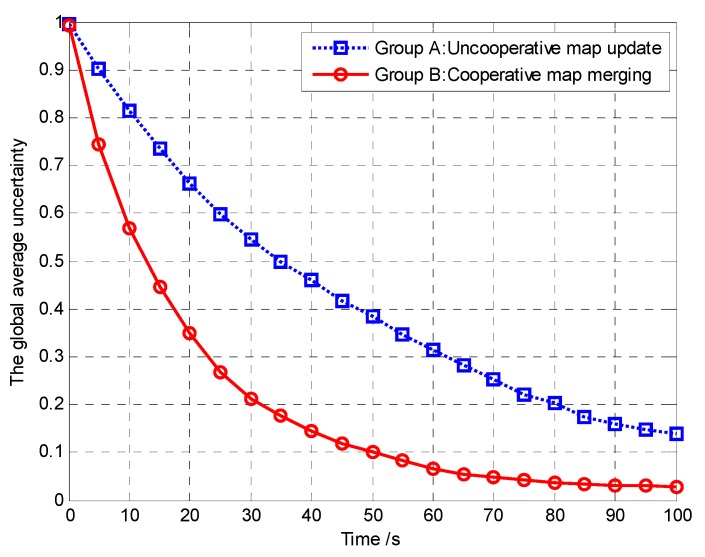
Comparison of the global average uncertainties (Scenario 7).

**Figure 38:**
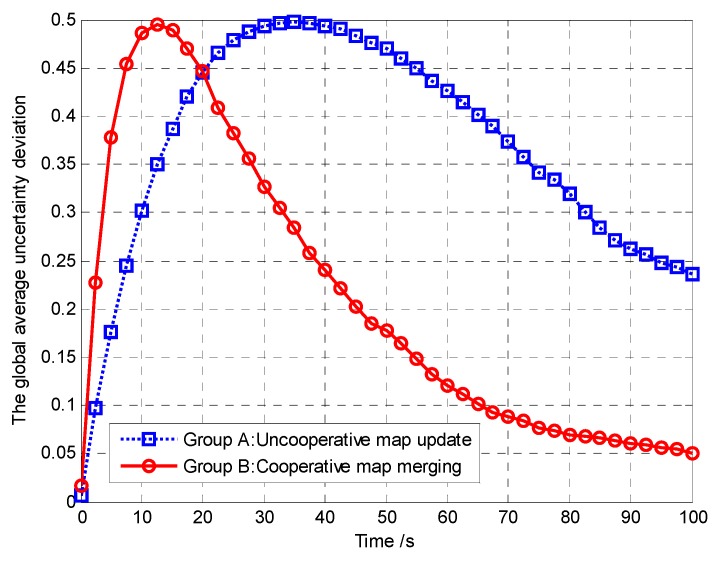
Comparison of the global average uncertainty deviations (Scenario 7).

We analyze and compare the global average uncertainty and the global average uncertainty deviation in Groups A and B, as shown in Figure 37 and Figure 38, respectively.

From Figure 37, it can be seen that the average uncertainty converges faster using our proposed distributed update and fusion scheme than by using the uncooperative map update method. This is because, if the neighboring UAVs exchange their current observations with *A_i_*, *A_i_* can get more observations each time than in the case without exchanging the observations, which increases the global average detected rates (*m_c_*_,*k*_/(*Nk*)) over the covered cells. Specifically, in addition to the observations taken over the cells within its own sensing range, the UAV *A_i_* can also get the observations taken over the cells outside its field of view (FoV) but inside the FoV of its neighbors. According to Theorem 3, the convergence speed of the cognitive maps in the UAVs is higher. The performance of mission operation is better with a higher convergence speed.

From Figure 38, it can be seen that, implementing the distributed update and fusion scheme, the average uncertainty deviation can decrease faster to 0. This is because the deviation between individual probability maps can be eliminated by exchanging the TPM for map fusion. Eventually, all individual target probability maps can converge to the same one, which reflects the existence or absence of the targets within each cell. However, in the uncooperative map update method, each UAV only updates its own TPM according to its sensor observations. In this case, it is hard to guarantee consensus among UAVs to maintain similar target probability maps and thus lead to mission performance degradations.

## 6. Conclusions

This paper mainly studies cooperative search and coverage for a given bounded rectangle region by a team of UAVs with non-ideal sensors and limited communication ranges. The main contribution of this paper is to develop a distributed cooperative search and coverage algorithm, which generates paths to gather more information about the environment and find more targets. Following conclusions can be obtained.

1. By integrating TPM, UM, and DPM, the cognitive map can effectively represent targets existence, uncertainty, and revisiting requirement of each cell in the surveillance region. Thus, the cognitive map can serve as the UAV’s knowledge of the environment, effectively.
2. Based on Bayesian rule and consensus theory, we design an update and fusion scheme of the TPM. We prove that the designed update and fusion scheme can guarantee each one of the TPMs converges to the same one that reflects the true environment, and the convergence speed of the TMP is proportional to the average detected rate of the cells in the surveillance region. This conclusion can provide theoretical guidance for the controllable revisit mechanism.
3. A controllable revisit mechanism based on the digital pheromone is proposed to control the UAVs to revisit some important areas that have high target probabilities or have not been explored for a long time. The results of comparison simulations show that the controllable revisit mechanism could enhance the capacities of target capture and region coverage for the UAVs compared to the method that does not consider the controllable revisit mechanism.
4. In path planning process, the movement of UAVs is restricted by the potential fields to meet the requirements of avoiding collision and maintaining connectivity constraints. Moreover, using the minimum spanning tree (MST) topology optimization strategy, we can obtain the tradeoff between the search coverage enhancement and the connectivity maintenance. The results of comparison simulations show that removing the redundant communication links may relax the motion restriction of multi-UAVs and improve the efficiency of cooperative search and coverage operation.

In future work, we will extend the cooperative search and coverage algorithm for moving targets and heterogeneous sensors. The impact of communication delays will also be considered, which will result in the deviation of individual cognitive maps.

## Figures and Tables

**Table 1:** The initial settings of 4 UAVs in Scenario 1.

UAVs *A_i_*	Position (*x_i_*, *y_i_*)/m	Occupant Cell (*m*, *n*)	Heading *ψ_i_*/(°)
*A*_1_	(−620, −980)	(10, 1)	45
*A*_2_	(−220, −980)	(20, 1)	135
*A*_3_	(−180, −980)	(30, 1)	45
*A*_4_	(580, −980)	(40, 1)	135

**Table 2:** The initial settings of 3 targets in Scenario 1.

Targets *T_j_*	Position (*x_j_*, *y_j_*)/m	Occupant Cell (*m*, *n*)
*T*_1_	(−620, −620)	(10, 10)
*T*_2_	(−620, 580)	(10, 40)
*T*_3_	(580, −620)	(40, 10)

## Appendix A. Proof of Theorem 1

Proof: Assume that, up to time *k*, the number of observations that have taken over cell *c* is *m_i,c,k_*, in which the number of *Z_i,c,k_* = 1 (i.e., “target detection”) is *a_i,c,k_*, the number of *Z_i,c,k_* = 0 (i.e., “non-target detection”) is (*m_i,c,k_* − *a_i,c,k_*). From Equation (9), we obtain

(A1) $$Q_{i,c,k}=Q_{i,c,0}+a_{i,c,k}\ln\frac{p_{f}}{p_{d}}+(m_{i,c,k}-a_{i,c,k})\ln\frac{1-p_{f}}{1-p_{d}}$$

If a target is present in cell *c* (i.e., *ζ_c_* = 1), *Z_i,c,k_* is a random variable based on the existence of targets, i.e., *P*(*Z_i,c,k_* = 1|*ζ_c_* = 1) = *p*_d_ and *P*(*Z_i,c,k_* = 0|*ζ_c_* = 1) = 1 − *p*_d_. In addition, *Z_i,c,k_* for all *k* > 0 are independent and identically distributed (i.i.d.) random variables. Then, according to the law of large numbers, as *m_i,c,k_* → +∞, (*a_i,c,k_*/*m_i,c,k_*) → *p*_d_. Thus, we get

(A2) $$\frac{Q_{i,c,k}}{m_{i,c,k}}=\frac{Q_{i,c,0}}{m_{i,c,k}}+\frac{a_{i,c,k}}{m_{i,c,k}}\ln\frac{p_{f}}{p_{d}}+(1-\frac{a_{i,c,k}}{m_{i,c,k}})\ln\frac{1-p_{f}}{1-p_{d}}\to \left(p_{d}\ln\frac{p_{f}}{p_{d}}+(1-p_{d})\ln\frac{1-p_{f}}{1-p_{d}}\right)<0$$

Hence, *Q_i,c,k_* → −∞(i.e., *p_i,c,k_* → 1) as *m_i,c,k_* → +∞. In the same way, we can prove the conclusion for no target presenting in cell *c* (i.e., *ζ_c_* = 0).

## Appendix B. Proof of Theorem 3

Proof: Let ***γ****_c,k_* = [*Q*_1,*c*,*k*_, …, *Q_N,c,k_*]^T^, ***V****_c,k_* = [*v*_1,*c*,*k*_, …, *v_N,c,k_*]^T^, [***W****_k_*]*_i, j_* = *ω_i,j,k_*; the distributed update and fusion rule in Equation (13) can be rewritten as

***γ**_c,k_* = ***W**_k_*(***γ***_*c,k* − 1_ + ***V**_c,k_*) (A3)

Additionally, we define $m_{c,k}=\sum_{i=1}^{N}m_{i,c,k}$ and $Q_{c,k}=\sum_{i=1}^{N}Q_{i,c,k}$.

Assume that, up to time *k*, the number of observations that have taken over cell *c* by *A_i_* is *m_i,c,k_*, in which the number of *Z_i,c,k_* = 1 (i.e., “target detection”) is *a_i,c,k_*, and the number of *Z_i,c,k_* = 0 (i.e., “non-target detection”) is (*m_i,c,k_* − *a_i,c,k_*). Then, we get

(A4) $$\sum_{l=1}^{k}\upsilon_{i,c,k}=a_{i,c,k}\ln\frac{p_{f}}{p_{d}}+(m_{i,c,k}-a_{i,c,k})\ln\frac{1-p_{f}}{1-p_{d}}$$

Therefore, for the whole team of UAVs, we have

(A5) $$Q_{c,k}=\sum_{i=1}^{N}Q_{i,c,k}=\sum_{i=1}^{N}Q_{i,c,0}+a_{c,k}ln\frac{p_{f}}{p_{d}}+(m_{c,k}-a_{c,k})\ln\frac{1-p_{f}}{1-p_{d}}$$

in which *a_c,k_* = $\sum_{i=1}^{N}a_{i,c,k}$ is the total number of “target detection” observations obtained by all UAVs up to time *k*. According to Theorem 1, we conclude that, if a target is present in the cell *c* (i.e., *ζ_c_* = 1), as *m_c,k_* → +∞, *Q_c,k_* → −∞ and

(A6) $$\frac{Q_{c,k}}{m_{c,k}}\to p_{d}\ln\frac{p_{f}}{p_{d}}+(1-p_{d})\ln\frac{1-p_{f}}{1-p_{d}}$$

Equation (A6) implies that, if each UAV can exchange the sensor observations with all the other UAVs, there is no deviation between each two individual target probability maps. All of the target probability maps converge to the same one, which reflects the targets’ true existence or absence in each cell of the search region. However, in our distributed update and fusion rule (Equation (13)), each UAV exchanges the target probability maps only with its neighboring UAVs. In this case, the deviation of the target probability maps exits. Next, we will prove that the deviation of the target probability maps is bounded by implementing the consensus protocol of Equation (13).

At time *k*, the deviations between all the target probability maps ***γ****_g,k_* and the global averaged probability map (((1/*N*)*Q_c,k_*)**1**) can be defined as

(A7) $$e_{c,k}=\gamma_{c,k}-\frac{1}{N}Q_{c,k}1=\gamma_{c,k}-\frac{1}{N}\left(1^{T}\gamma_{c,k}\right)1$$

in which **1** = [1] _*N*×1_ = [1, 1, …, 1]^T^. Therefore, according Equations (A3) and (A7), we have

(A8) $$\left\|e_{c,k}\right\|=\left\|\prod_{t=1}^{k}W_{t}\gamma_{c,0}-\frac{1}{N}1^{T}\left(\prod_{t=1}^{k}W_{t}\right)\gamma_{c,0}1+\sum_{l=1}^{k}\prod_{t=l}^{k}W_{t}V_{c,l}-\frac{1}{N}1^{T}\left(\sum_{l=1}^{k}\prod_{t=l}^{k}W_{t}V_{c,l}\right)1\right\|$$

Due to **1**^T^ ***W****_k_* = **1**^T^, ***W****_k_***1** = **1** and [***W****_k_*]*_i,j_* ≥ 0 for all *i* and *j*, we get

(A9) $$\left\|e_{c,k}\right\|=\left\|\Delta_{\;1,\;k}+\Delta_{2,k}\right\|\leq \left\|\Delta_{1,k}\right\|+\left\|\Delta_{2,k}\right\|$$

(A10) $$\Delta_{1,k}=\prod_{t=1}^{k}W_{t}\gamma_{c,0}-\frac{1}{N}\left(1^{T}\gamma_{c,0}\right)1,\Delta_{2,k}=\sum_{l=1}^{k}\left(\prod_{t=l}^{k}W_{t}V_{c,l}-\frac{1}{N}\left(1^{T}V_{c,l}\right)1\right)$$

According to the consensus theory, it can be easily proved that, given any (*N* × 1) vector ***θ*** and any (*N* × *N*) matrix ***W****_k_* that is associated with a connected topology ***G***(*k*), we can find a number 0 < *λ* < 1 such that the following inequality holds.

(A11) $$\left\|W_{k}\theta -\frac{1}{N}\left(1^{T}\theta \right)1\right\|\leq \lambda \left\|\theta -\frac{1}{N}\left(1^{T}\theta \right)1\right\|$$

Therefore, we have

(A12) $$\left\|\Delta_{\;1,\;k}\right\|\leq \lambda^{k}\left\|\gamma_{c,0}-\frac{1}{N}\left(1^{T}\gamma_{c,0}\right)1\right\|$$

(A13) $$\left\|\Delta_{2,k}\right\|\leq \sum_{l=1}^{k}\left(\left\|\prod_{t=l}^{k}W_{t}V_{c,l}-\frac{1}{N}\left(1^{T}V_{c,l}\right)1\right\|\right)\leq \sum_{l=1}^{k}\left(\lambda^{k-l+1}\left\|V_{c,l}-\frac{1}{N}\left(1^{T}V_{c,l}\right)1\right\|\right)$$

Substituting Equations (A12) and (A13) into Equation (A9), we get

(A14) $$\left\|e_{c,k}\right\|\leq \lambda^{k}\left\|\gamma_{c,0}-\frac{1}{N}\left(1^{T}\gamma_{c,0}\right)1\right\|+\sum_{l=1}^{k}\left(\lambda^{k-l+1}\left\|V_{c,l}-\frac{1}{N}\left(1^{T}V_{c,l}\right)1\right\|\right)$$

According to Equation (9), it is easy to get

(A15) $$\left\|V_{c,l}-\frac{1}{N}\left(1^{T}V_{c,l}\right)1\right\|<\sqrt{N}\left|\ln\frac{p_{f}}{p_{d}}-\ln\frac{1-p_{f}}{1-p_{d}}\right|$$

Substituting Equation (A15) into Equation (A14), we get

(A16) $$\left\|e_{c,k}\right\|\leq \lambda^{k}\left\|\gamma_{c,0}-\frac{1}{N}\left(1^{T}\gamma_{c,0}\right)1\right\|+\left(\sqrt{N}\left|\ln\frac{p_{f}}{p_{d}}-\ln\frac{1-p_{f}}{1-p_{d}}\right|\right)\left(\frac{\lambda (1-\lambda^{k})}{1-\lambda }\right)$$

Thus, we have

(A17) $$\underset{m_{c,k}\to +\infty }{\lim}\left\|e_{c,k}\right\|=\underset{k\to +\infty }{\lim}\left\|e_{c,k}\right\|<\left|\ln\frac{p_{f}}{p_{d}}-\ln\frac{1-p_{f}}{1-p_{d}}\right|\left(\frac{\lambda \sqrt{N}}{1-\lambda }\right)$$

Equation (A17) implies that the deviations between all the individual target probability maps and the globally averaged map are bounded. Thus, we have

(A18) $$\frac{\gamma_{c,k}}{m_{c,k}}\to \frac{1}{N}\left(\frac{Q_{c,k}}{m_{c,k}}\right)1$$

Substituting Equation (A6) into Equation (A18), we get

(A19) $$\frac{\gamma_{c,k}}{m_{c,k}}\to \frac{1}{N}\left(\frac{Q_{c,k}}{m_{c,k}}\right)1\to \frac{1}{N}\left(p_{d}\ln\frac{p_{f}}{p_{d}}+(1-p_{d})\ln\frac{1-p_{f}}{1-p_{d}}\right)1$$

which implies

(A20) $$\frac{Q_{i,c,k}}{m_{c,k}}\to \frac{1}{N}\left(p_{d}\ln\frac{p_{f}}{p_{d}}+(1-p_{d})\ln\frac{1-p_{f}}{1-p_{d}}\right)$$

In other words, if a target is present in the cell *c* (i.e., *ζ_c_* = 1), as *m_c,k_* → +∞, then *Q_i,c,k_* → −∞ (i.e., *p_i,c,k_* → 1) and (*Q_i,c,k_*/*m_c,kk_*) → (*p*_d_/*N*)ln(*p*_f_/*p*_d_) + ((1 − *p*_d_)/*N*)ln(1 − *p*_f_/1 − *p*_d_). In the same way, we can prove the conclusion for no target presenting in cell *c* (i.e., *ζ_c_* = 0).
